# Targeted blocking of CCR2 and CXCR2 improves the efficacy of transarterial chemoembolization of hepatocarcinoma

**DOI:** 10.1186/s12935-022-02771-z

**Published:** 2022-11-19

**Authors:** Zhiqiang Tian, Xiaojuan Hou, Wenting Liu, Changchun Shao, Lu Gao, Jinghua Jiang, Li Zhang, Zhipeng Han, Lixin Wei

**Affiliations:** 1 Tumor Immunology and Gene Therapy Center, Third Affiliated Hospital of Naval Medical University, Shanghai, 200438 China; 2 National Center for Liver Cancer, Shanghai, China; 3 Clinical Research Unit, The First Affiliated Hospital of Naval Medical University, Shanghai, China; 4 Department of General Surgery, The Affiliated Wuxi People’s Hospital of Nanjing Medical University, Wuxi, China

## Abstract

**Background:**

Transarterial chemoembolization (TACE) has been shown to prolong survival in patients with unresectable hepatocellular carcinoma (HCC); however, the long-term survival remains dismal. Targeting macrophage and neutrophil infiltration is a promising strategy. The CCL2/CCR2 and CXCLs/CXCR2 axes are required for recruitment of macrophages and neutrophils, respectively, in HCC. We investigated the feasibility of CCL2/CCR2 and CXCLs/CXCR2 as therapeutic targets in combination with TACE for treating HCC.

**Methods:**

Expression of CCL2/CCR2 and CXCLs/CXCR2 was analyzed in the primary rat HCC model and one HCC cohort. The relationship between expression levels, neutrophil and macrophage infiltration, hepatocarcinogenesis progression in the rat model, and survival of HCC patients was assessed. The anti-tumor effects of blocking the CCL2/CCR2 and CXCLs/CXCR2 axes by CCR2 and CXCR2 antagonists in combination with TACE were evaluated in HCC rats. The numbers of macrophages, neutrophils, and hepatic progenitor cells were further determined to explore the underlying mechanisms.

**Results:**

High macrophage and neutrophil infiltration and CXCL8 expression were associated with poor prognosis in the TCGA liver cancer dataset. High expression of CCL2/CCR2 and CXCL8/CXCR2 in clinical HCC specimens was associated with reduced survival. Expression of CCL2/CCR2 and CXCL1/CXCR2 was correlated with hepatocarcinogenesis progression in the primary rat HCC model. Blockade of CCL2/CCR2 and CXCLs/CXCR2 enhanced the anti-tumor effect of TACE treatment in this model. Blocking the CCL2/CCR2 and CXCLs/CXCR2 axes with CCR2 and CXCR2 antagonists in TACE-treated rats reduced macrophage and neutrophil infiltration and hepatic progenitor cell activation and thus overcame TACE resistance in HCC.

**Conclusions:**

The results demonstrate the translational potential of immunotherapy targeting the CCL2/CCR2 and CXCLs/CXCR2 axes in combination with TACE therapy for the treatment of HCC.

**Supplementary Information:**

The online version contains supplementary material available at 10.1186/s12935-022-02771-z.

## Background

Hepatocellular carcinoma (HCC), a common and usually lethal type of liver cancer, is the third leading cause of cancer-related death worldwide [1]. Although the clinical treatment of HCC has been improving [2], the overall treatment effect is always unsatisfactory and has not fundamentally changed the high mortality rate of HCC [3]. The poor prognosis of HCC is due to aggressive malignant potential including invasive and metastatic activity.

Transarterial chemoembolization (TACE) has become an accepted, standard treatment in the management of patients with intermediate-stage HCC according to the Barcelona Clinic Liver Cancer algorithm [4, 5]. TACE in the setting of primary and secondary HCC is also becoming an essential part of the oncology landscape [6]. While TACE is effective in killing HCC cells in both theoretical and in vitro experiments, there is an urgent need to identify novel therapeutic strategies for TACE due to the poor prognosis of patients receiving this procedure [7]. The major problems in the clinical application of TACE include unclear mechanisms of resistance to TACE treatment, lack of prognostic indicators for effectiveness/resistance, and possible lack of active ingredients in available embolic agents [8]. It is essential to thoroughly understand the mechanism of TACE resistance in HCC both in vitro and in vivo.

HCC is an inflammation-related cancer, and inflammation is an important feature of the tumor microenvironment [9]. Chronic liver injury triggered by hepatocellular carcinogenic factors is mainly mediated by uncontrollable chronic inflammation, with a large number of macrophages, neutrophils, T cells, and natural killer cells infiltrating the liver. Increased infiltration of immune cells in the tumor microenvironment is involved in the development and progression of hepatocellular carcinoma through the secretion of large amounts of inflammatory factors that induce immune-mediated hepatocellular injury [10]. The presence of an inflammatory microenvironment in hepatocellular carcinoma tissues is considered to be an important influencing factor in the recurrence and metastasis of HCC [11].

The chemokine receptor CCR2 with its ligand CCL2, as well as the receptor CXCR2 and its multiple ligands CXCL1, CXCL2, and CXCL8, have been implicated in a wide range of tumors [12, 13]. There is extensive evidence to indicate that the CCL2/CCR2 and CXCLs/CXCR2 axes can stimulate tumor growth and angiogenesis, and promote the infiltration and activation of host immune cells [14]. The CCL2/CCR2 axis is involved in the recruitment of monocytes and macrophages to tumor sites [13]. Recent studies indicated that the CXCLs/CXCR2 axis contributes to tumor progression in HCC through the infiltration of neutrophils and myeloid-derived suppressor cells [15, 16]. The CCL2/CCR2 and CXCL8/CXCR2 axes play an essential role in the malignant progression of tumors and act as novel targets for cancer treatment [17]. The importance of the CCL2/CCR2 and CXCL8/CXCR2 axes in various cancers has led to the development of several small-molecule inhibitors that target the two axes [18, 19]. Therefore, we specifically focused on the involvement of these two chemokine networks in promoting the recruitment of immune cells and increasing angiogenesis, tumor growth, and metastasis in HCC.

Based on the existing evidence, we hypothesized that blocking the CCL2/CCR2 and CXCLs/CXCR2 axes would enhance the TACE-induced inhibition of tumor growth. The aims of the present study, involving the use of a primary rat HCC model and retrospective cohort studies of patients with HCC, were (1) to investigate the role of the CCL2/CCR2 and CXCLs/CXCR2 axes in HCC tumor growth and progression, (2) to evaluate the effect of targeting the CCL2/CCR2 and CXCLs/CXCR2 axes on TACE-induced antitumor activity, and (3) to investigate the underlying mechanism.

## Methods

### Bioinformatics analysis

The impact of immune infiltration on patient overall survival (OS) in The Cancer Genome Atlas (TCGA) liver cancer dataset was evaluated by the TIMER 2.0 web server [20]. Due to the limitations of direct measurement methods, computational algorithms are often used to infer immune cell composition from bulk tumor transcriptome profiles. TIMER2.0 is a comprehensive resource for systematical analysis of the abundances of different immune infiltrates across 32 cancer types from TCGA. Outcome module of TIMER2.0 allows users to explore the clinical relevance of tumor immune subsets. In this database we explored the correlation between six tumor immune subsets (CD8^+^ T cells, CD4^+^ T cells, B cells, Neutrphils, Macrophages and Dendritic cells) and survival rate of HCC patients. The Kaplan–Meier plots were drawn with TIMER2.0 for immune infiltrates and HCC to visualize the survival differences. The log-rank *p*—value for Kaplan–Meier curves are shown in the figures. Immune cells infiltrate levels are divided into low- and high- level groups by the split percentage of patients 50%.

The expression of CCL2/CCR2 and CXCL8/CXCR2 in HCC tissues was explored with RNA sequencing data in the TCGA liver cancer dataset, and analyzed by the UALCAN web server (http://ualcan.path.uab.edu/index.html) [21]. Patients were stratified according to CCL2/CCR2 and CXCL8/CXCR2 expression values in HCC: High (> 3rd quartile), Medium (between 3rd and 1st quartile), Low (< 1st quartile). Survival rates were analyzed by Kaplan–Meier plots. Significance of survival impact was measured by log rank test. A *p*-value < 0.05 was considered statistically significant.

The correlations between CCL2/CCR2 and CXCLs/CXCR2 gene expression and immune infiltrates were evaluated by the TIMER 2.0 program of the TCGA liver cancer dataset [20]. Gene module of TIMER2.0 allows users to select any gene of interest and visualize the correlation of its expression with immune infiltration level in diverse cancer types. In simple terms, gene module draws an association between known instances of expression of a gene in an immune cell type versus a tumor type and therefore concludes that expression of that gene is therefore either positively correlated with infiltration of a particular type of immune cell if the gene is known to be expressed in that immune cell or negatively correlated if it is more commonly expressed in tumors. Scatter plots were used to examine the relationship between gene expression levels and the infiltration levels of immune cells, as estimated by the TIMER algorithm based on RNA-Seq expression profile data.

The UCSC Xena browser (https://xenabrowser.net) [22] was utilized to obtain the corresponding violin plots of the mRNA expression levels of CCL2/CCR2 and CXCL8/CXCR2 between tumor and adjacent normal tissues, based on TCGA liver cancer data.

### Patient cohort and liver specimens

Liver specimens were obtained from 74 HCC patients who accepted hepatectomy in the Shanghai Eastern Hepatobiliary Surgery Hospital from 1996 to 2006. We retrospectively collected the clinicopathological and follow-up data of patients. These patients included 67 men and 7 women, with a median age of 50.5 ± 12.4 years. All the liver specimens were subjected to immunohistochemistry analysis. Prior informed consent was obtained. The human studies were approved by the Ethics Boards of Shanghai Eastern Hepatobiliary Surgery Hospital and written informed consent was obtained from each patient. The detailed demographic and clinicopathological characteristics of those 74 HCC patients undergoing hepatectomy are summarized in Table 1.

**Table 1:** Demographic and Clinicopathological Characteristics of 74 HCC Patients

Factors	Value	Percent
Age	50.5 ± 12.4	
Gender (male/female)	67/7	90.5/9.5
BCLC Stage		
A	2	2.7
B	34	45.9
C	38	51.4
Types of cirrhosis		
Normal	17	23.3
Micronodular	4	5.4
Macronodular	34	45.9
Mixed	19	25.7
TNM stage		
1	32	43.2
2	36	48.7
3	6	8.1
Neoadjuvant Therapy	0	0
Hepatectomy	74	100
Laboratory values		
White blood cell (× 10^9^/L)	5.48 ± 1.99	
Platelet (× 10^9^/L)	146 ± 73	
Total bilirubin (umol/L)	14.6 ± 5.5	
Direct bilirubin (umol/L)	5.6 ± 2.7	
Albumin (g/L)	42.6 ± 4.1	
ALT (U/L)	56.1 ± 40.5	
AST (U/L)	42.7 ± 21.4	
Glucose (mmol/L)	5.0 ± 1.0	
CEA (ng/ml)	4.2 ± 7.9	
CA19-9 (U/ml)	14.9 ± 8.5	
AFP (ng/ml)	324.7 ± 425.3	

*AFP* alpha-fetoprotein, *ALT* alanine aminotransferase, *AST* aspartate aminotransferase, *BCLC* the Barcelona Clinic Liver Cancer, *CA19-9* carbohydrate antigen 19–9, *CEA* Carcinoembryonic antigen, *TNM* tumor-node-metastasis

### Primary rat HCC model

To establish a primary rat HCC model, male Sprague Dawley rats (8–10 weeks old, weighing 160–180 g) were purchased from Shanghai Laboratory Animal Research Center (Shanghai, China). The rats were housed under specific pathogen-free conditions. Rats received diethylnitrosamine through drinking water (concentration of 100 ppm) to induce liver cancer. More than 90% of the diethylnitrosamine-treated rats developed macroscopic liver tumors (multiple, diameter > 2 mm) at 12–14 weeks. Animal experiments were approved by the Ethics Board of Second Military Medical University. All procedures were performed following the institutional animal welfare guidelines of Second Military Medical University.

### Inhibition of the CCL2/CCR2 and CXCL1/CXCR2 axes in HCC rats

Sprague Dawley rats with diethylnitrosamine induced HCC were used to simulate human liver cancer. INCB3344 is a selective CCR2 antagonist which blocks the CCL2/CCR2 axis. SCH527123 is a selective, small-molecule CXCR2 antagonist which blocks the CXCL1/CXCR2 axis. After diethylnitrosamine treatment for 6 weeks, the rats were divided into 4 treatment groups consisting of 10 animals per group. The rats were anesthetized with 2% sevoflurane, then intraperitoneally injected with INCB3344 (60 μg/g body weight) in 200 μl saline, or SCH527123 (10 μg/g body weight) in 200 μl saline, or INCB3344 (60 μg/g) + SCH527123 (10 μg/g) in 200 μl saline, or 200 μl of blank saline. Injections were repeated twice every week for 7 weeks. After the whole treatment schedule was complete (13 weeks), the rats were sacrificed to observe the development of HCC.

### TACE regimens

After 14 weeks of diethylnitrosamine treatment, Sprague Dawley rats were randomized into five groups, with ten rats in each group. As described below, cisplatin-based TACE was performed in the five groups with or without INCB3344 and SCH527123. We found that the 3 mg/kg dose of cisplatin was well tolerated by the rat. A surface area-based conversion factor ('FDA Guidance for Industry: Estimating the Maximum Safe Starting Dose in Initial Clinical Trials for Therapeutics in Adult Healthy Volunteers’, available at https://www.fda.gov/media/72309/download) predicts the rat 3 mg/kg dose to be equivalent to a 18 mg/m^2^ dose for a 60 kg human, and this dose is equally to the clinically recommended dose, just above the lower end of the 18–30 mg/m^2^ dosing range recommended by the Chinese clinical practice guidelines for TACE of HCC. The treatment groups were as follows: I: control group (0.5 ml normal saline); II: cisplatin (3 mg/kg); III: cisplatin (3 mg/kg) plus INCB3344 (60 μg/g); IV: cisplatin (3 mg/kg) plus SCH527123 (10 μg/g); and V: cisplatin (3 mg/kg) plus INCB3344 (60 μg/g) and SCH527123 (10 μg/g).

### TACE therapy in the primary HCC model

Sprague Dawley rats with diethylnitrosamine induced HCC were used to simulate human liver cancer. TACE was performed in rats after 14 weeks of diethylnitrosamine treatment. Briefly, a polyethylene catheter (PE-10) with a 0.6-mm outside diameter connected to one end of a needle (inner diameter: 0.2 mm) was used for catheterization under laparotomy. The rat was anesthetized with 2% sevoflurane and then immobilized on a foam board. The rat was initially scraped clean and the liver was exposed using an abdominal median incision. After exposure of the liver, the needle was inserted retrogradely into the gastroduodenal artery using a binocular surgical microscope. Successful intubation was confirmed by a perfusion measurement. The above-mentioned agents were injected through the catheter into the hepatic artery at a speed of about 0.1 ml/3 min, followed by with 0.1 ml of normal saline. The hepatic artery was then ligated with 5–0 silk thread. The ventral incision was closed with a permanent suture in two layers. The rats were sacrificed 6 weeks after administration of the different treatments.

### Histological examination

Rat liver tissue specimens were removed and fixed in 10% phosphate-buffered formalin for 24 h. The liver cancer specimens were embedded in paraffin, sectioned to 4 mm thickness, and stained with H&E for histological examination. The Klintrup-Makinen score was used to quantify the peritumoral inflammatory infiltrate in H&E sections. The density of the generalized inflammatory cell infiltrate was graded as low-grade (no or mild increase in inflammation) and high grade (prominent increase in inflammation with cancer cell destruction) as previously described [23]. Each sample was assessed and scored independently by two pathologists who were blinded to the study protocol.

### Immunohistochemical staining

The sample staining procedures were performed as previously described [24]. The following antibodies were used for immunocytochemistry: anti-CCL2 (1:200 dilution, cat. no. DF7577, Affinity, USA), anti-CCR2 (1:200 dilution, cat. no. 357207, Biolegend, San Diego, CA, USA), anti-CXCL8 (1:250 dilution, cat. no. ab7747, Abcam, Cambridge, UK), and anti-CXCR2 (1:200 dilution, cat. no. 320705, Biolegend, San Diego, CA, USA), anti-CD68 (1:200 dilution, cat. no. ab31630, Abcam, Cambridge, UK), anti-iNOS (1:500 dilution, cat. no. ab178945, Abcam, Cambridge, UK), anti-CD163 (1:500 dilution, cat. no. ab182422, Abcam, Cambridge, UK), anti-MPO (1:200 dilution, cat. no. GB11224, Servicebio, Wuhan, China), and anti-CK19 (1:1000 dilution, cat. no. 14965–1-AP, Proteintech Group, Rosemont, IL). Phosphate buffered saline was used in negative controls. Kaplan–Meier estimated of overall survival in 74 patients with HCC according to the expression level of CCL2, CCR2, CXCL8 or CXCR2 in IHC. Images of five randomly chosen fields per slide were taken with a microscope (× 200 magnification). Image-Pro Plus 6.2 was employed to analyse the levels of protein expression by calculating the values of mean integrated optical density (integrated optical density/area) for statistical analysis [9]. Patients were divided into low- and high-expression groups by the median of mean integrated optical density.

### Statistical analysis

All experiments were repeated at least 3 times with similar results. All quantitative data are expressed as mean ± SD for each experiment. Comparisons between two experimental groups were made using Student’s t-test. One-way analysis of variance was performed using GraphPad Prism software version 7.0. Analysis of clinical data was performed with SPSS Statistics software version 24.0 (IBM Corporation, New York, NY, U.S.A.). Relationships between continuous variables were analyzed using Pearson’s correlation coefficient (r) and Spearman’s rank correlation coefficient (rho). Survival times were analyzed by Kaplan–Meier survival analysis with the log-rank test for comparison. Statistically significant differences are indicated as follows: *p < 0.05, **p < 0.01, and ***p < 0.001.

## Results

### High neutrophil and macrophage infiltration and CXCL8 expression are associated with low survival

We summarized the HCC-associated chemokines and their receptors of previous studies, and focused on their target immune cell subpopulations (Additional file 1: Table S1). The results show that a total of 32 chemokines and 19 chemokine receptors are closely associated with HCC, and the main relevant immune cell subsets include CD4^+^ T cells, CD8^+^ T cells, B cells, neutrophils, macrophages, and dendritic cells (Additional file 1: Table S1). TIMER 2.0 is a comprehensive resource for systematic analysis of immune infiltrates across diverse cancer types. This version of the webserver provides information on the abundance of infiltrating immune cells, estimated by multiple immune deconvolution methods, and allows users to comprehensively explore tumor immunological, clinical and genomic features. We investigated the correlation between hepatocellular carcinoma-associated chemokines and their receptors and the level of immune infiltration in HCC using TIMER 2.0. Results for the six above-identified immune cell types are presented in Additional file 1: Tables S2–S7. To make the results clearer and more visual, we integrated the separate datasets into a single table for analysis (Additional file 1: Table S8). The results showed that the expression levels of most hepatocellular carcinoma-associated chemokines and their receptors were positively correlated with the degree of immune cell infiltration in HCC. A single chemokine (CCL14) was negatively correlated, while a few chemokines (CCL25, CCL27 and CXCL2) were not correlated (Additional file 1: Table S8).

We explored the correlation between the level of tumor immune subpopulation infiltration and clinical prognosis in the TCGA liver cancer database using TIMER 2.0 (Additional file 1: Fig. S1A–F). The results showed that the infiltration levels of CD8^+^ T cells, CD4^+^ T cells, B cells, and dendritic cells (DCs) did not correlate with the overall survival (OS) of HCC patients, whereas the infiltration levels of neutrophils and macrophages did correlate with HCC OS. When the degree of neutrophil and macrophage infiltration was low, the cumulative survival rate of hepatocellular carcinoma patients was increased. We then explored the HCC tumor stage relevance of tumor immune subsets in a multivariable Cox proportional hazard model. The results showed that CD8^+^ T cells had no significant effect on survival time (P = 0.918), stage 3 (HR = 2.68, 95%CI 1.75–4.09, P < 0.0001) and stage 4 (HR = 5.49, 95%CI 1.69–17.82, P = 0.005) patients had a statistically significant impact on survival time; CD4^+^ T cells had no significant effect on survival time (P = 0.566), stage 3 (HR = 2.67, 95%CI 1.74–4.05, P < 0.0001) and stage 4 (HR = 5.31, 95%CI 1.63–17.31, P = 0.006) patients had a statistically significant impact on survival time; B cells had no significant effect on survival time (P = 0.431), stage 3 (HR = 2.66, 95%CI 1.74–4.06, P < 0.0001) and stage 4 (HR = 5.22, 95%CI 1.60–17.04, P = 0.006) patients had a statistically significant impact on survival time; Neutrophil had no significant effect on survival time (P = 0.247), stage 3 (HR = 2.58, 95%CI 1.68–3.96, P < 0.0001) and stage 4 (HR = 4.58, 95%CI 1.35–15.50, P = 0.015) patients had a statistically significant impact on survival time; Macrophage had significant effect on survival time (P = 0.002), stage 3 (HR = 2.58, 95%CI 1.68–3.96, P < 0.0001) and stage 4 (HR = 6.09, 95%CI 1.87–19.82, P = 0.003) patients had a statistically significant impact on survival time; DCs had no significant effect on survival time (P = 0.104), stage 3 (HR = 2.65, 95%CI 1.74–4.05, P < 0.0001) and stage 4 (HR = 5.66, 95%CI 1.74–18.37, P = 0.004) patients had a statistically significant impact on survival time. Through a comprehensive analysis of the results in Additional file 1: Table S1, Additional file 1: Table S8 and Additional file 1: Fig. S1, we found that the CCL2/CCR2 (macrophage-related) and CXCL8/CXCR2 (neutrophil-related) axes are closely associated with the prognosis of HCC patients and can be used as new therapeutic targets for hepatocellular carcinoma.

Therefore, we performed a more detailed analysis of the role of these axes in hepatocellular carcinoma. The association between the expression of genes in the CCL2/CCR2 and CXCL8/CXCR2 axes and patient OS in the TCGA liver cancer dataset was analyzed. Kaplan–Meier survival curves were constructed using the UALCAN browser. In this cohort, although the expression levels of CCL2, CCR2, and CXCR2 were not significantly associated with poor prognosis, HCC patients with high expression of CXCL8 had shorter OS compared to the patients with low expression (Additional file 1: Fig. S1G).

We further investigated the correlation between the expression levels of the CCL2/CCR2 and CXCLs/CXCR2 genes with the immune infiltration level in HCC. Scatter plots were constructed using the TIMER 2.0 browser based on RNA sequencing expression profile data. The analysis was performed on five genes (CCL2, CCR2, CXCL1, CXCL8, and CXCR2) and the estimated infiltration levels of six immune cell types (CD8^+^ T cells, CD4^+^ T cells, B cells, DCs, neutrophils, and macrophages). The results showed a positive correlation between the gene expression level and immune cell infiltration in HCC (*p* < 0.05), except for CXCL1 and CD8^+^ T cells, CXCR2 and CD4^+^ T cells, and CXCR2 and B cells (Additional file 1: Fig. S2). Therefore, targeting CCL2/CCR2 and CXCLs/CXCR2 can potentially regulate immune cell infiltration in the tumor microenvironment and improve the therapeutic effect of HCC.

Collectively, these results suggest that reducing neutrophil and macrophage infiltration improves OS in patients with HCC, and that targeting CCL2/CCR2 and CXCL8/CXCR2 chemotaxis is feasible as a potential therapeutic strategy.

### High expression of CCL2/CCR2 and CXCL8/CXCR2 in HCC specimens is associated with low survival

**Fig. 1:**
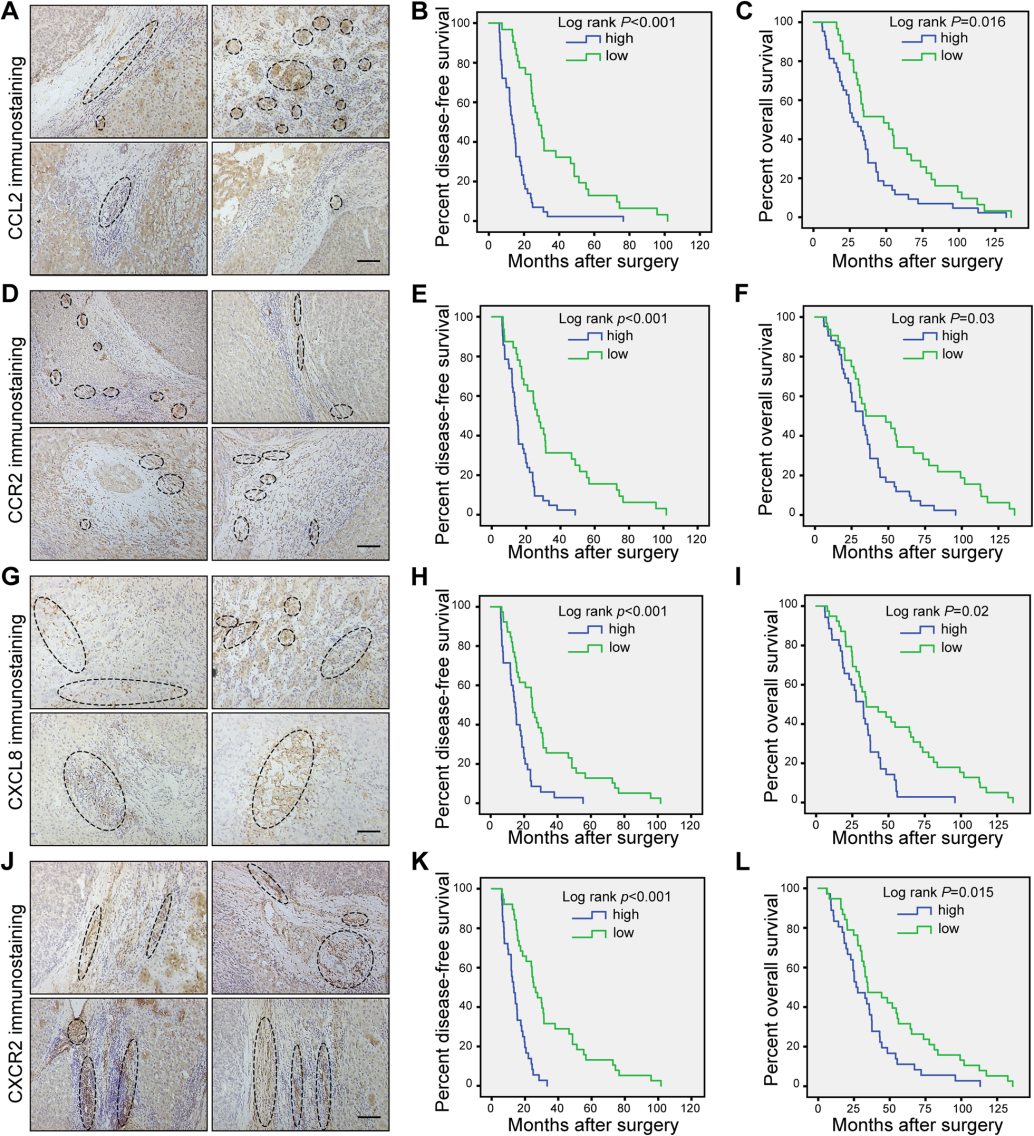
High expression levels of CCL2/CCR2 and CXCL8/CXCR2 in HCC specimens are associated with poor prognosis. Kaplan–Meier estimated of overall survival in 74 patients with HCC according to the expression level of CCL2, CCR2, CXCL8 and CXCR2 in immunohistochemical. Images of five randomly chosen fields per slide were taken with a microscope. Image-Pro Plus 6.2 was employed to analyse the levels of protein expression by calculating the values of mean integrated optical density (integrated optical density/area) for statistical analysis. Patients were divided into low- and high-expression groups by the median of mean integrated optical density. **A** Four representative images of CCL2 immunostaining in clinical HCC specimens. **B**, **C** The Kaplan–Meier curves for disease-free survival (DFS) and overall survival (OS) of this cohort according to low and high expression of CCL2. **D** Four representative images of CCR2 immunostaining in clinical HCC specimens. **E**–**F** Kaplan–Meier curves for DFS and OS according to the expression levels of CCR2. **G** Four representative images of CXCL8 immunostaining in clinical HCC specimens. **H**–**I** Kaplan–Meier DFS and OS curves according to the expression levels of CXCL8. **J** Four representative images of CXCR2 immunostaining in clinical HCC specimens. **K**–**L** Kaplan–Meier DFS and OS curves according to the expression levels of CXCR2. The log-rank *p*-value for Kaplan–Meier curve is shown on the Kaplan–Meier curve plot. *p* < 0.05 was considered as statistically significant. Scale bar, 200 μm

To explore the role of the CCL2/CCR2 and CXCL8/CXCR2 axes in HCC, expression of CCL2, CCR2, CXCL8, and CXCR2 was evaluated by immunohistochemistry in tissue microarrays of tumors from a retrospective cohort of 74 HCC specimens with complete follow-up data. Typical images of CCL2, CCR2, CXCL8, and CXCR2 immunostaining in clinical HCC samples are shown in Fig. 1. Moreover, Kaplan–Meier survival analysis was performed on the association between protein expression levels (CCL2, CCR2, CXCL8, and CXCR2) and disease-free survival (DFS) and overall survival (OS) (Fig. 1). There was an inverse association between the CCL2, CCR2, CXCL8 and CXCR2 protein levels and DFS (all log-rank *p* < 0.001). Likewise, we found a significant inverse association between CCL2, CCR2, CXCL8 and CXCR2 protein levels and OS (log-rank *p* = 0.016, *p* = 0.03, *p* = 0.02 and *p* = 0.015, respectively) (Fig. 1). Our results showed that high levels of CCL2, CCR2, CXCL8, and CXCR2 in HCC tissues were correlated with poor prognosis and reduced patient survival.

To further confirm these results, we compared the expression levels of the CCL2/CCR2 and CXCL8/CXCR2 genes in HCC tumors and adjacent normal tissues using data from the TCGA liver cancer database. The RNA expression levels were visualized with violin plots, obtained via the UCSC Xena browser (https://xenabrowser.net). Significant differences in expression levels were observed between tumors and adjacent normal tissues in HCC cohorts (Additional file 1: Fig. S3). The results showed that CCL2, CCR2, CXCL8, and CXCR2 were more highly expressed in adjacent normal tissues than in liver cancer tissues. The above results suggest that high expression of CCL2/CCR2 and CXCL8/CXCR2 in the peri-carcinoma tissue can mediate the interaction between immune cells and liver cancer cells and promote the development of HCC.

### CCL2/CCR2 and CXCL1/CXCR2 expression levels are correlated with hepatocarcinogenesis progression in primary HCC model

**Fig. 2:**
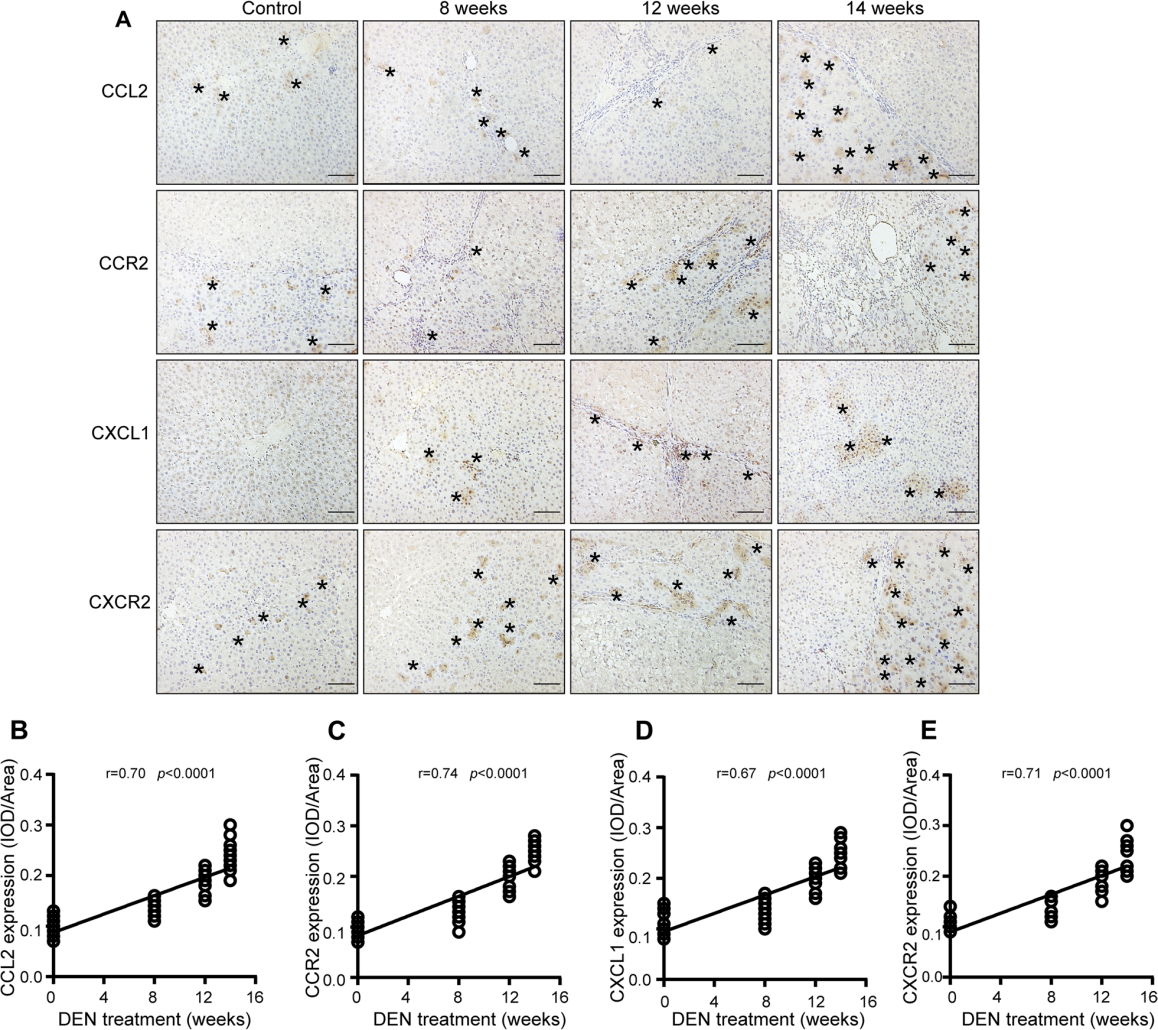
Expression levels of CCL2/CCR2 and CXCL1/CXCR2 are correlated with HCC progression in the HCC model. Sprague Dawley rats were treated with diethylnitrosamine. After 8, 12 or 14 weeks, the rats were sacrificed to observe the development of HCC. The expression levels of CCL2, CCR2, CXCL1, and CXCR2 proteins were detected by immunohistochemical analysis. As CXCL8 is absent in rodents, we examined CXCL1, which is considered the functional equivalent of human CXCL8 in promoting neutrophil migration. The integral optical density (IOD) of the stained sections was calculated, and then Pearson’s correlation analysis was performed to investigate the relationship between the protein expression level and diethylnitrosamine treatment time. Pearson’s correlation analysis yielded the correlation coefficient (*r*) and the *p*-value. **A** Representative images of CCL2, CCR2, CXCL1, and CXCR2 immunostaining in the liver tissue of rats after 8, 12 and 14 weeks of diethylnitrosamine treatment. Asterisks indicate positive staining. Scale bar 200 μm. (B-E) Pearson's correlation analysis between **B** CCL2 and diethylnitrosamine treatment time (r = 0.70, p < 0.0001), **C** CCR2 and diethylnitrosamine treatment time (r = 0.74, p < 0.0001), **D** CXCL1 and diethylnitrosamine treatment time (r = 0.67, p < 0.0001), and **E** CCR2 and diethylnitrosamine treatment time (r = 0.71, p < 0.0001)

The CXCL8 gene is absent in rodents, so we examined CXCL1 in rats, which is considered the functional equivalent of human CXCL8 in promoting neutrophil migration [25]. To explore the role of the CCL2/CCR2 and CXCL1/CXCR2 axes in primary HCC, expression of CCL2, CCR2, CXCL1, and CXCR2 was evaluated by immunohistochemical analysis of tissue microarrays of tumors from the primary rat HCC model at different time points (Fig. 2A). The expression of CCL2, CCR2, CXCL1, and CXCR2 was enhanced during hepatocarcinogenesis progression. There was a significant positive correlation between CCL2, CCR2, CXCL1, CXCR2 expression and diethylnitrosamine treatment time in the primary rat HCC model (r = 0.70, *p* < 0.0001; r = 0.74, *p* < 0.0001; r = 0.67, *p* < 0.0001; r = 0.71, *p* < 0.0001; respectively) (Fig. 2B–E). The results indicate that the CCL2/CCR2 and CXCLs/CXCR2 axes play an important role in HCC progression.

Based on their polarization states, macrophages are divided into three types: general macrophages (M0 macrophages), classically activated type 1 (M1 macrophages), and alternatively activated type 2 (M2 macrophages) [26]. M1 macrophages can produce and release proinflammatory mediators. In contrast, M2 macrophages often result in the synthesis of anti-inflammatory cytokines and contribute to the resolution of inflammation. To explore whether the three different macrophage activation states are correlated with hepatocarcinogenesis over time in the primary rat HCC model, the expression of CD68 (M0 macrophage marker), iNOS (M1 macrophage marker), and CD163 (M2 macrophage marker) were detected by immunohistochemical analysis at different times point (Additional file 1: Fig. S4A). The expression levels of CD68 and CD163 were augmented during HCC progression. However, the expression level of iNOS was attenuated. There was a significant positive correlation between CD68, iNOS and CD163 expression and diethylnitrosamine treatment time in the primary rat HCC model (r = 0.09, *p* = 0.01; r = 0.21, *p* < 0.0001; r = 0.60, *p* < 0.0001; respectively) (Additional file 1: Fig. S4B-D). The results show that during the occurrence and development of HCC, although the total number of macrophages did not change significantly, their polarized phenotypic subpopulations did change significantly. M1-type macrophages with anti-tumor effects gradually decreased, while M2-type macrophages with tumor-promoting effects gradually increased.

### Intraperitoneal CCR2 and CXCR2 antagonists prevent hepatocarcinogenesis in the primary HCC model

**Fig. 3:**
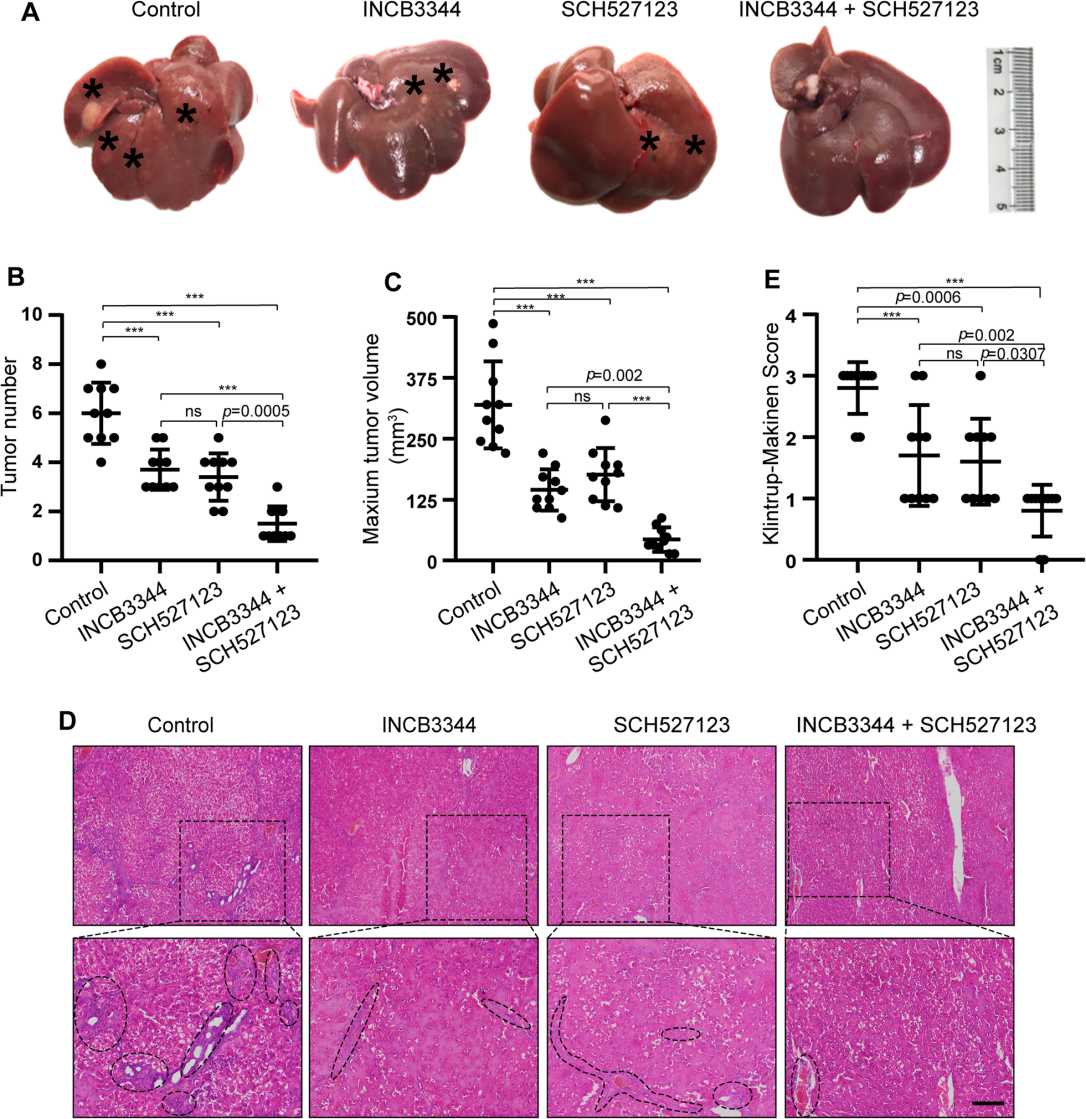
Intraperitoneal administration of CCR2 and CXCR2 antagonists prevents hepatocarcinogenesis in the primary HCC model. **A** After 8 weeks of diethylnitrosamine treatment, Sprague Dawley rats were intraperitoneally injected with the CCR2 antagonist INCB3344 (60 μg/g body weight) in 200 μl saline, the CXCR2 antagonist SCH527123 (10 μg/g body weight) in 200 μl saline, INCB3344 (60 μg/g) + SCH527123 (10 μg/g) in 200 μl saline, or 200 μl of blank saline. Injections were continued twice every week for 6 weeks. After 14 weeks, the rats were sacrificed to observe the development of HCC. Representative images of rat livers from the indicated treatment groups are shown (n = 10/group). Typical tumor nodes are marked by the asterisks. **B** The number of HCC nodules per liver was counted. Data are presented as mean ± SD. *ns* not statistically significant, ***p < 0.001. **C** The maximum tumor volume per liver was determined. Data are presented as mean ± SD. *ns* not statistically significant, ***p < 0.001. **D** Hematoxylin and eosin **H**, **E** staining was employed to observe the histological structure of the liver in the indicated treatment groups. Black asterisks represent accumulation of inflammatory cells. Scale bar, 200 μm. **E** The Klintrup-Makinen score was determined to assess the level of local inflammatory cell infiltration. Data are presented as mean ± SD. *ns* not statistically significant, ***p < 0.001

**Fig. 4:**
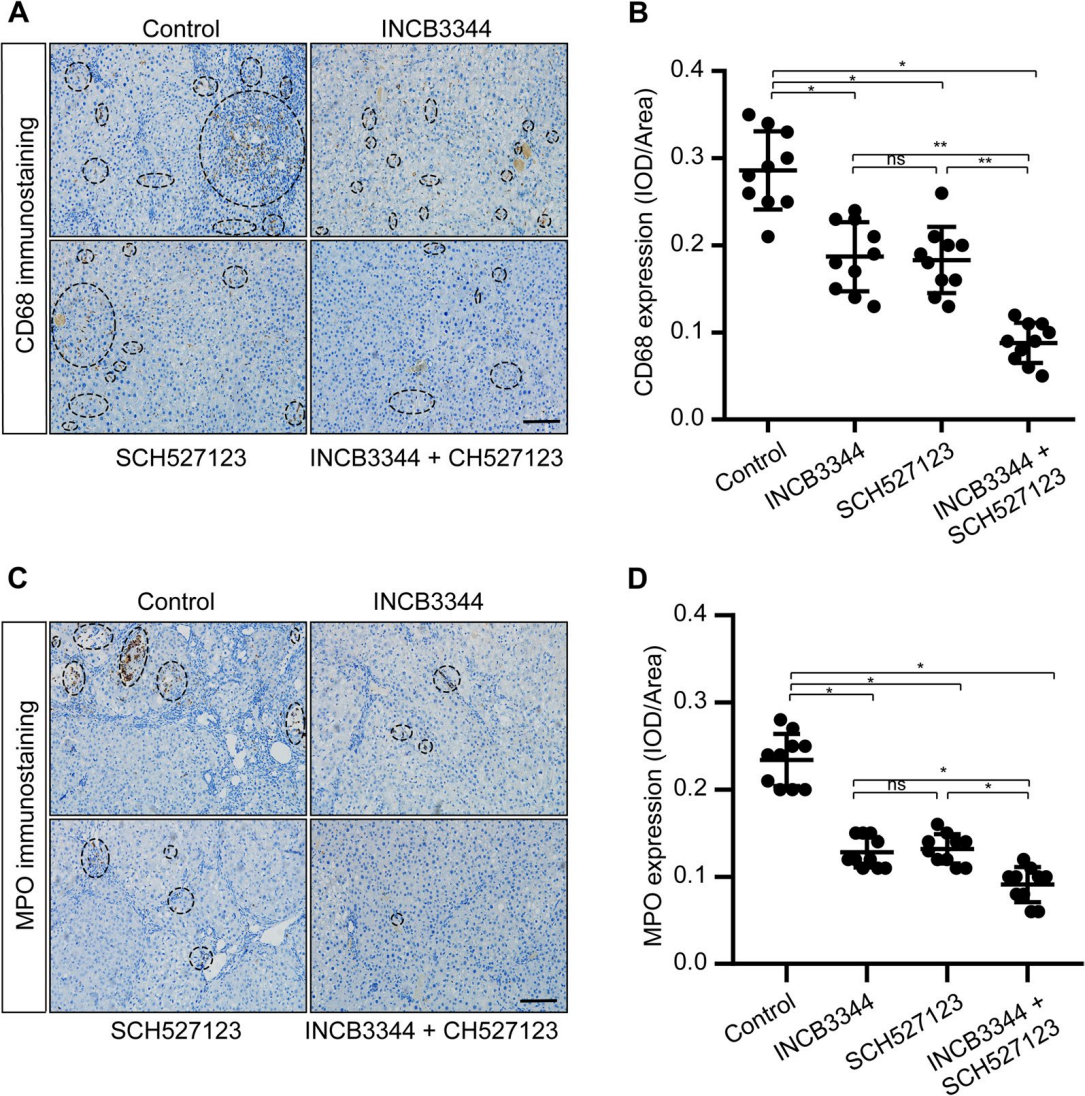
CCR2 and CXCR2 antagonists reduce the recruitment of TAMs/TANs in the primary HCC model. Sprague Dawley rats were treated with diethylnitrosamine for 14 weeks, then INCB334 and SCH527123 were administered via intraperitoneal injection. After 14 weeks, the rats were sacrificed and the livers were removed for immunohistochemical detection of CD68 and MPO. CD68 is a specific marker for TAMs while MPO is a specific marker for TANs. The expression levels of CD68 and MPO in the stained sections was assessed by IOD. **A**, **C** Representative images of CD68 and MPO immunostaining in livers from the indicated groups. Asterisks indicate positive staining. Scale bar, 200 μm. **B** Comparison of CD68 levels, based on IOD, of the indicated groups. **D** Comparison of MPO levels, based on IOD, of the indicated groups. Data are presented as mean ± SD. *ns* not statistically significant, *p < 0.05, **p < 0.01

Due to the contribution of the CCL2/CCR2 and CXCL1/CXCR2 axes to the pathology of various cancers, there have been attempts to develop CCR2 and CXCR2 antagonists, anticipating therapeutic benefits from CCR2 and CXCR2 inhibition. Here, we evaluated the feasibility of targeting CCR2 and CXCR2 with their respective antagonists INCB3344 and SCH527123 for liver cancer therapy. We assessed the antitumor effect of intraperitoneal injection of CCR2 and CXCR2 antagonists in the rat primary HCC model. The rats showed the overall tolerability to the used therapies we found the general health of rats is good in treated and vehicle groups. Representative images of HCC tumors in the different treatment groups are shown in Fig. 3. Compared to the control group, the number of tumors and the maximum tumor volume were reduced following intraperitoneal injection of CCR2 and CXCR2 antagonists for 6 weeks (Fig. 3A, C). Measurement of the body weight was used to evaluate the health status of the rats after therapy treatment. The rats were in good general health status and their body weights increased in the treated animals versus control animals as displayed in Additional file 1: Fig. 5. The results from hematoxylin and eosin (H&E) staining and Klintrup-Makinen scoring showed that intraperitoneal injection of CCR2 and CXCR2 antagonists reduced the inflammatory response in the liver (Fig. 3D, E). Immunohistochemical staining also showed that the groups receiving CCR2 and CXCR2 antagonists had significantly lower levels of CD68 and myeloperoxidase (MPO) expression, which indicates that the antagonists reduced the number of neutrophils and macrophages in HCC tissues (Fig. 4). These results suggest that CCR2 and CXCR2 antagonists can inhibit the development of liver cancer by inhibiting the inflammatory response.

### Blockade of CCL2/CCR2 and CXCL1/CXCR2 enhances the antitumor effect of TACE in the primary HCC model

The TACE procedure was modified for use in the rat primary HCC model (Additional file 1: Fig. S6A). Briefly, a polyethylene catheter with a 0.6-mm outside diameter was connected to one end of a needle (inner diameter: 0.2 mm) and used for catheterization under laparotomy. After exposure of the liver, the needle was inserted retrogradely into the gastroduodenal artery using a binocular surgical microscope. The chemotherapeutic drug cisplatin (3 mg/kg body weight) was injected through the catheter into the hepatic artery. Control animals were administered 0.5 ml normal saline. The hepatic artery was then ligated (Additional file 1: Fig. S6B). The rats showed the overall tolerability to the used therapies we found the general health of rats is good in treated and vehicle groups. Representative images of rat livers, including typical tumor nodes, from the control and TACE treatment groups are shown in Additional file 1: Fig. S6C. The tumor numbers following cisplatin-based TACE 6 weeks in HCC rats were reduced compared to the control group. These results indicate that the cisplatin-based TACE treatment reduced the number of tumor nodes in the primary rat HCC model.

**Fig. 5:**
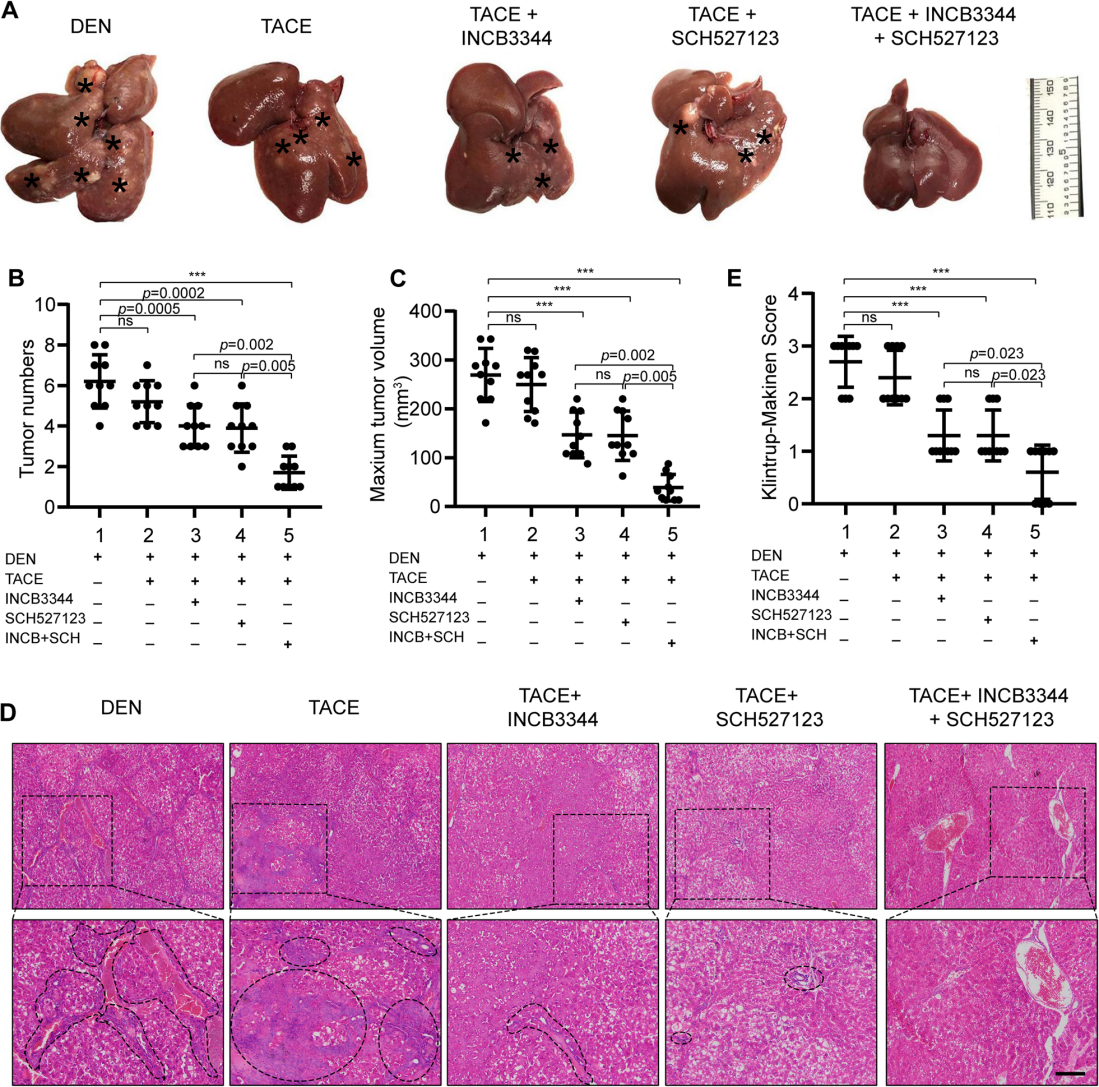
Blockade of CCL2/CCR2 and CXCL1/CXCR2 enhances the antitumor effect of TACE in the HCC model. **A** Sprague Dawley rats were treated with diethylnitrosamine for 14 weeks, then subjected to the modified TACE procedure. The chemotherapeutic drug cisplatin (3 mg/kg body weight) was administered to the TACE-treated rats with or without the CCR2 antagonist INCB3344 (60 μg/g body weight) and the CXCR2 antagonist SCH527123 (10 μg/g body weight). Control animals received the TACE procedure and administered 0.5 ml normal saline. Representative images of rat livers from the indicated treatment groups are shown. Typical tumor nodes are indicated by the asterisks (n = 10/group). **B** The number of HCC nodules per liver. Data are presented as mean ± SD. ns, not statistically significant, ***p < 0.001. **C** The maximum tumor volume per liver. Data are presented as mean ± SD. ns, not statistically significant, ***p < 0.001. **D** Hematoxylin and eosin **H**, **E** staining was employed to observe the histological structure of the liver in the indicated groups. Black asterisks represent accumulation of inflammatory cells. Scale bar, 200 μm. **E** The Klintrup-Makinen score was determined to assess the level of local inflammatory cell infiltration. Data are presented as mean ± SD. *ns* not statistically significant, ***p < 0.001

**Fig. 6:**
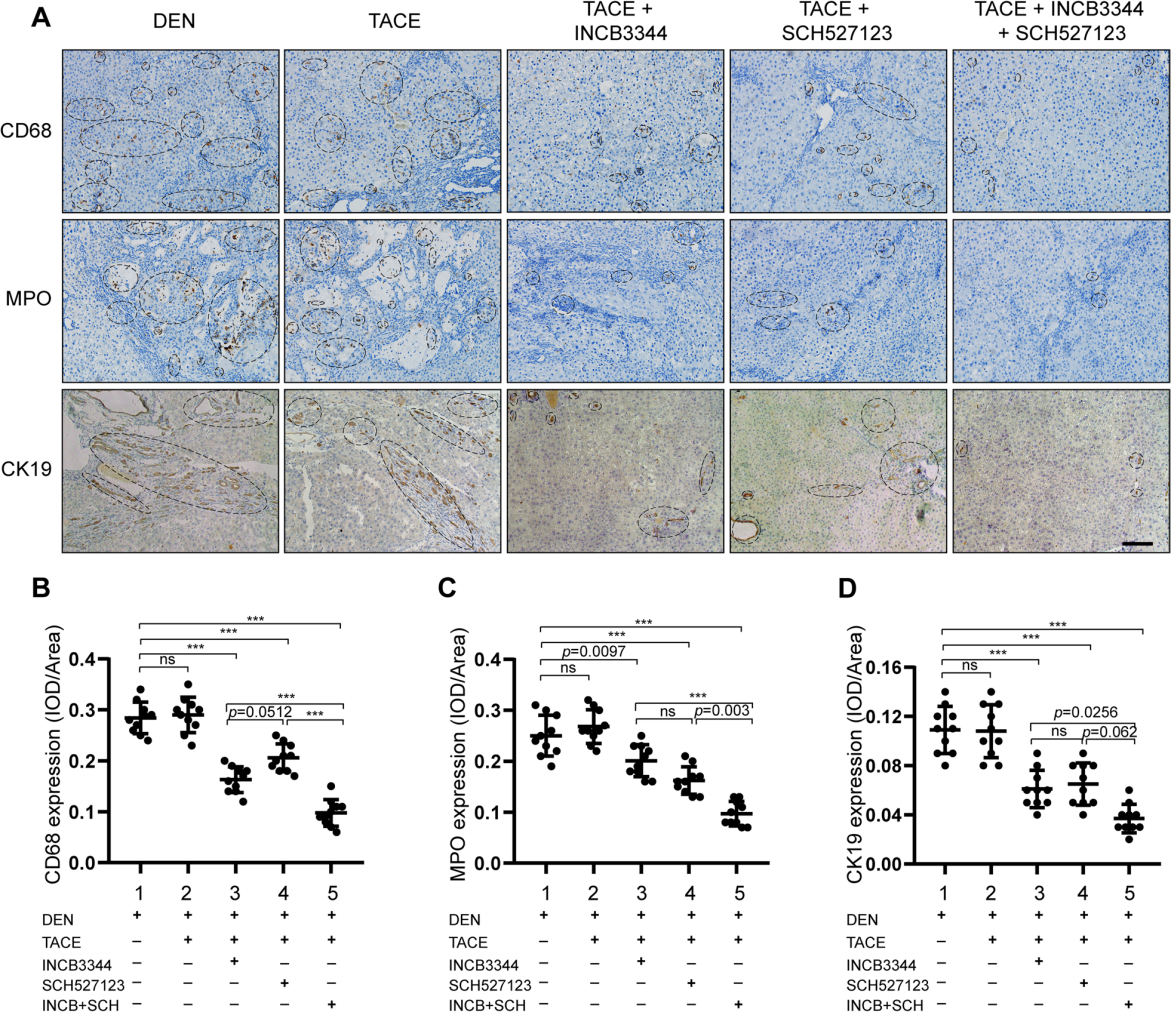
CCR2 and CXCR2 antagonists inhibited HCC by reducing neutrophils and macrophages infiltration and HPCs activation. Rats were treated as in Fig. 5. Liver sections were prepared for immunohistochemical detection of CD68, MPO, and CK19. CK19 is a specific marker for hepatic progenitor cells. The positive area of each slice was quantified as average IOD with Image-Pro Plus software. **A** Representative images of CD68, MPO, and CK19 immunostaining in rat livers of the indicated groups. Scale bar, 200 μm. **B**–**D** Comparison of **B** CD68 levels, **C** MPO levels, and **D** CK19 levels of the indicated groups based on IOD results. Data are presented as mean ± SD. *ns* not statistically significant, ***p < 0.001

Next, we clarified the role of CCR2 and CXCR2 antagonists in the TACE-induced inhibition of tumor growth. HCC was induced in rats with diethylnitrosamine for 14 weeks, then the rats were subjected to TACE and cisplatin with or without INCB3344 and SCH527123. As shown in Fig. 5, the CCR2 and CXCR2 antagonists significantly suppressed liver tumor growth when combined with TACE. Representative images of livers, including HCC tumors, from the different treatment groups are shown in Fig. 5A. The tumor numbers and the maximum tumor volume following CCR2 and CXCR2 antagonists-based TACE 6 weeks in HCC rats were reduced compared to the control group (Fig. 5B, C). Measurement of the body weight was used to evaluate the health status of the rats after therapy treatment. The rats were in good general health status and their body weights increased in the treated animals versus control animals as displayed in Additional file 1: Fig. S7. The results from H&E staining and Klintrup-Makinen scoring showed that TACE combined with the CCR2 and CXCR2 antagonists reduced the inflammatory response in the livers (Fig. 5D–E). CK19 was known to be a specific marker for hepatic progenitor cells. In addition, immunohistochemical staining showed that the group receiving TACE plus CCR2 and CXCR2 antagonists had significantly lower levels of CD68, MPO and CK19 expression (Fig. 6). These results indicate that TACE combined with CCR2 and CXCR2 antagonists reduced neutrophils and macrophages recruitment and hepatic progenitor cell activation.

## Discussion

Our study assessed the relationship between the expression of CCL2/CCR2 and CXCLs/CXCR2, neutrophil and macrophage infiltration, hepatocarcinogenesis progression in a rat model, and survival of HCC patients. Based on the significant association between the two chemokine axes and HCC, we hypothesized that targeted therapies against CCL2/CCR2 and CXCLs/CXCR2 can improve the anti-tumor effect of TACE. The main findings of this study are: (1) high neutrophil and macrophage infiltration and CXCL8 expression are associated with poor prognosis in the TCGA liver cancer dataset; (2) high expression of CCL2/CCR2 and CXCL8/CXCR2 in clinical HCC specimens is associated with poor prognosis; (3) the expression levels of CCL2/CCR2 and CXCL1/CXCR2 are correlated with hepatocarcinogenesis progression in the primary rat HCC model; and (4) blockade of CCL2/CCR2 and CXCL1/CXCR2 enhance the anti-tumor effect of TACE in the primary rat HCC model.

In the clinic, TACE is recommended for treatment of unresectable HCC, as it is able to deprive the tumor of its blood supply, thus inducing ischemic necrosis [27–29]. However, numerous previous studies have proposed that complete tumor necrosis is seldom achieved after TACE and most of the tumor nodules contain viable HCC cells at the necrotic margin [30, 31]. HCC is the archetype of inflammation-associated cancers [32]. Infiltration of immune cells following TACE is considered to serve an important role in modulating the hepatic microenvironment [33]. Neutrophils and macrophages are primary subsets of cells that infiltrate the tumor microenvironment, and have been implicated as essential drivers of tumor progression, which leads to failure of TACE treatment [34, 35]. Therefore, therapeutic strategies for inhibiting neutrophil and macrophage infiltration may improve the outcomes of HCC patients receiving TACE [36, 37]. In this study, we found that high neutrophil and macrophage infiltration was associated with poor postoperative survival rate in HCC. We reviewed recent findings on immune cell infiltration and related chemokine expression in the HCC microenvironment. The evidence allowed us to conclude that macrophage and neutrophil infiltration are closely associated with HCC through CCL2/CCR2 and CXCLs/CXCR2, respectively. To validate these findings, we further confirmed that high expression levels of CCL2/CCR2 and CXCL8/CXCR2 were associated with poor prognosis in clinical HCC specimens and correlated with hepatocarcinogenesis progression in the primary rat HCC model.

In recent years, immunotherapy that relies on the composition of the tumor microenvironment has become increasingly popular [38, 39]. However, the unique immune response in the liver favors tolerance, which represents a substantial challenge for conventional immunotherapy in patients with HCC [40]. Regulation of the immune microenvironment by targeting the chemokine families would be of great clinical benefit [41]. As a step towards this goal, we therefore investigated the function of CCR2 and CXCR2 antagonists in HCC rats receiving TACE treatment. Our results show that treatment with CCR2 and CXCR2 antagonists improves the effect of TACE to provide increased anti-tumor activity.

Tumors are very complex and it is difficult to find cellular or animal models to perfectly mimic them. Studies targeting the immune microenvironment also need to elucidate the characteristics of tumors and the surrounding immune cells, etc. In vitro, tumor-derived organoids are commonly used models to study the interaction of tumor-like bodies with fibroblasts, mesenchymal cells, and immune cells in the microenvironment [42]. In vivo, the orthotopic xenograft model is a classic animal model, in which the injection frequency and number of transplanted cells can be adjusted to create a temporary tumor environment in situ [43]. However, the disadvantage is that this model is usually constructed in immunosuppressed mice and therefore lacks active immune cells in the tumor microenvironment. diethylnitrosamine is a well-known carcinogen to study HCC in rodent models. In this study we induced HCC by diethylnitrosamine in Sprague Dawley rats. The advantage of the diethylnitrosamine induced HCC rat model is the resemblance of gene expression patterns and the tumor microenvironment to HCC in humans [44]. We then established TACE in the diethylnitrosamine induced HCC rat model, and found that it well simulated the TACE procedure in HCC patients. We showed that targeting the CCL2/CCR2 and CXCLs/CXCR2 axes in rats overcomes the resistance of HCC to TACE. Moreover, we made a preliminary exploration of the mechanism underlying the effect of the CCR2 and CXCR2 antagonists. Our results show that targeting the CCL2/CCR2 and CXCLs/CXCR2 axes in the context of TACE treatment reduces the infiltration of neutrophils and macrophages and the activation of hepatic progenitor cells, thus overcoming the TACE resistance of HCC.

## Conclusions

In summary, we have uncovered a new therapeutic strategy for targeting neutrophil and macrophage infiltration in TACE therapy for HCC via inhibition of the CCL2/CCR2 and CXCLs/CXCR2 axes. Blocking these axes with CCR2 and CXCR2 antagonists in the context of TACE reduces neutrophil and macrophage infiltration and hepatic progenitor cell activation, thus overcoming the TACE resistance of HCC. This finding will further facilitate our adoption of immunotherapy targeting the CCL2/CCR2 and CXCLs/CXCR2 axes in combination with TACE for the treatment of patients with advanced HCC.

## Supplementary Information

**Additional file 1:** **Figure S1.** Neutrophil and macrophage infiltration and CXCL8expression are associated with poor prognosis. （A-F）TIMER2.0 is a comprehensive database for systematical analysis of the abundances of different immune infiltrates across 32 cancer types from TCGA. We explored the correlation between six immune subsets (CD8+ T cells, CD4+ T cells, B cells, Neutrphils, Macrophages and Dendritic cells) and survival rate of HCC patients. The Kaplan- Meier plots were drawn with TIMER2.0 for immune infiltrates and HCC to visualize the survival differences. Immune cells infiltrate levels are divided into low- and high- level groups by the split percentage of patients 50%. The hazard ratio (HR) for the Cox model and the log-rank p-value for Kaplan–Meier curves are shown. (G) The expression levels of CCL2, CCR2, CXCL8 and CXCR2 in HCC tissues were explored with RNA sequencing data from the TCGA liver cancer dataset, and analyzed by the UALCAN web server (http://ualcan.path.uab.edu/index.html). Kaplan-Meier OS curves are shown according to low and high expression of CCL2, CCR2, CXCL8, and CXCR2. p < 0.05 was considered as statistically significant. **Figure S2.** Correlation of CCL2/CCR2 and CXCLs/CXCR2 genes’ expression with immune infiltration level in HCC. The correlation between genes expression of CCL2/CCR2 and CXCLs/CXCR2 and immune infiltrates were evaluated via the TIMER 2.0 web server in the TCGA liver cancer cohort. TIMER2.0 is freely available at http://timer.cistrome.org. Scatter plots show the relationship between gene (CCL2, CCR2, CXCL1, CXCL8, and CXCR2) expression level and six infiltrating immune cells (CD8+ T cells, CD4+ T cells, B cells, dendritic cells, neutrophils, and macrophages) estimation value by TIMER algorithm based on RNA-Seq expression profiles data. **Figure S3.** Compare CCL2/CCR2 and CXCLs/CXCR2 genes’ expression of tumor and adjacent normal tissues in HCC. The UCSC Xena browser (https://xenabrowser.net) was utilized to obtain the corresponding violin plots of the mRNA expression levels of CCL2/CCR2 and CXCL8/CXCR2 between tumor and adjacent normal tissues of TCGA liver cancer database. (A) Different expression of CCL2 between HCC and adjacent normal tissues (*p *= 3.435e-14). (B) Different expression of CCR2 between HCC and adjacent normal tissues (*p *= 0.002). (C) Different expression of CXCL8 between HCC and adjacent normal tissues (*p *= 0.000008). (D) Different expression of CXCR2 between HCC and adjacent normal tissues (*p *= 1.953e-17). Primary tumor (n=371), adjacent normal tissue(n=50). Welch's t test, *p *value <0.05. **Figure S4.** Macrophage phenotypic switched correlated with hepatocarcinogenesis progression in primary rat HCC model. Sprague Dawley rats were treated with DEN. The rats were sacrificed to observe the development of HCC. To understand the phenotypes of infiltrated macrophages in rat hepatocarcinogenesis progression, the expression of CD68 (a general macrophage marker), iNOS (a surface marker for M1 macrophage phenotype), and CD163 (a surface marker for M2 macrophage phenotype) were detected by immunohistochemical. The stained sections were evaluated by IOD, and then the correlation of the gene expression level and DEN treatment times was analyzed by the Pearson’s correlation. Pearson’s correlation analysis provided correlation coefficient (*r*) and *p*-value. (A) Representative immunohistochemical images of CD68, iNOS, and CD163 staining in rat liver tissue of the indicated groups were shown. (B) Pearson's correlation analysis between CD68 and DEN treatment times (r = 0.09, p = 0.01). (C) Pearson's correlation analysis between iNOS and DEN treatment times (r = 0.21, p < 0.0001). (D) Pearson's correlation analysis between CD163 and DEN treatment times (r = 0.60, p < 0.0001). **Figure S5.** Intraperitoneal administration of CCR2 and CXCR2 antagonists prevents hepatocarcinogenesis in the primary HCC model. After 8 weeks of diethylnitrosamine (DEN) treatment, Sprague Dawley rats were intraperitoneally injected with the CCR2 antagonist INCB3344 (60 μg/g body weight) in 200 μl saline, the CXCR2 antagonist SCH527123 (10 μg/g body weight) in 200 μl saline, INCB3344 (60 μg/g) + SCH527123 (10 μg/g) in 200 μl saline, or 200 μl of blank saline. Injections were continued twice every week for 6 weeks. After 14 weeks, the rats were sacrificed to observe the development of HCC. The body weight of the rats was recorded. Data are presented as mean ± SD. **p < 0.01, ***p < 0.001. **Figure S6.** TACE in primary rat HCC model. (A) Sprague Dawley rats with DEN-induced HCC were used to simulate human liver cancer. TACE procedure was performed in rat after 14 weeks DEN treatment. (B) TACE procedure was modified in the primary rat HCC model. Briefly, a polyethylene catheter (PE-10) with a 0.6-mm outside diameter connected to one end of a needle (inner diameter: 0.2mm) was used for catheterization under laparotomy. After exposure of the liver tumor, the needle was inserted retrogradely into the gastroduodenal artery by using a binocular operative microscope. The mentioned agents were injected through the catheter to the hepatic artery. The hepatic artery was then ligated. (C) Representative images of rat livers of the control and TACE treatment group, the typical tumor nodes were shown by the asterisks. **Figure S7.** Blockade of CCL2/CCR2 and CXCL1/CXCR2 enhances the antitumor effect of TACE in the HCC model. Sprague Dawley rats were treated with diethylnitrosamine (DEN) for 14 weeks, then subjected to the modified TACE procedure. The chemotherapeutic drug cisplatin (3 mg/kg body weight) was administered to the TACE-treated rats with or without the CCR2 antagonist INCB3344 (60 μg/g body weight) and the CXCR2 antagonist SCH527123 (10 μg/g body weight). Control animals received the TACE procedure and administered 0.5ml normal saline. The body weight of the rats was recorded. Data are presented as mean ± SD. *ns* not statistically significant, *p < 0.05, **p < 0.01. **Table S1.** HCC-associated chemokine families and their functions in immune infiltrates. **Table S2.** The relationship between HCC-associated chemokine expression and the infiltrate estimation value for CD8+ T cells in the TCGA LIHC dataset. **Table S3.** The relationship between HCC-associated chemokines expression and the infiltrate estimation value for CD4+ T cells in the TCGA LIHC dataset. **Table S4.** The relationship between HCC-associated chemokine expression and the infiltrate estimation value for B cells in the TCGA LIHC dataset. **Table S5**. The relationship between HCC-associated chemokine expression and the infiltrate estimation value for neutrophils in the TCGA LIHC dataset. **Table S6.** The relationship between HCC-associated chemokine expression and infiltrate estimation value for macrophages in the TCGA LIHC dataset. **Table S7**. The relationship between HCC-associated chemokine expression and infiltrate estimation value for dendritic cells in the TCGA LIHC dataset. **Table S8**. The relationship between HCC-associated chemokine expression and immune infiltrate estimation values in the TCGA LIHC dataset.

## Data Availability

The data of this study are available from the corresponding author upon reasonable request.

## Abbreviations

TACE: Transarterial chemoembolization
HCC: Hepatocellular carcinoma
OS: Overall survival
TCGA: The Cancer Genome Atlas
IOD: Integrated optical density
DCs: Dendritic cells
DFS: Disease-free survival

## References

[1] Bray F, Ferlay J, Soerjomataram I, Siegel RL, Torre LA, Jemal A (2018). Global cancer statistics 2018: GLOBOCAN estimates of incidence and mortality worldwide for 36 cancers in 185 countries. CA Cancer J Clin.

[2] Yang JD, Hainaut P, Gores GJ, Amadou A, Plymoth A, Roberts LR (2019). A global view of hepatocellular carcinoma: trends, risk, prevention and management. Nat Rev Gastroenterol Hepatol.

[3] Yang JD, Heimbach JK (2020). New advances in the diagnosis and management of hepatocellular carcinoma. BMJ.

[4] Malagari K, Pomoni M, Kelekis A, Pomoni A, Dourakis S, Spyridopoulos T, Moschouris H, Emmanouil E, Rizos S, Kelekis D (2010). Prospective randomized comparison of chemoembolization with doxorubicin-eluting beads and bland embolization with BeadBlock for hepatocellular carcinoma. Cardiovasc Intervent Radiol.

[5] Forner A, Gilabert M, Bruix J, Raoul JL (2014). Treatment of intermediate-stage hepatocellular carcinoma. Nat Rev Clin Oncol.

[6] Habib A, Desai K, Hickey R, Thornburg B, Lewandowski R, Salem R (2015). Transarterial approaches to primary and secondary hepatic malignancies. Nat Rev Clin Oncol.

[7] Kudo M, Ueshima K, Ikeda M, Torimura T, Tanabe N, Aikata H, Izumi N, Yamasaki T, Nojiri S, Hino K (2020). Randomised, multicentre prospective trial of transarterial chemoembolisation (TACE) plus sorafenib as compared with TACE alone in patients with hepatocellular carcinoma: TACTICS trial. Gut.

[8] Wei X, Zhao L, Ren R, Ji F, Xue S, Zhang J, Liu Z, Ma Z, Wang XW, Wong L (2021). MiR-125b Loss Activated HIF1alpha/pAKT Loop, Leading to transarterial chemoembolization resistance in hepatocellular carcinoma. Hepatology.

[9] Liu WT, Jing YY, Gao L, Li R, Yang X, Pan XR, Yang Y, Meng Y, Hou XJ, Zhao QD (2020). Lipopolysaccharide induces the differentiation of hepatic progenitor cells into myofibroblasts constitutes the hepatocarcinogenesis-associated microenvironment. Cell Death Differ.

[10] Keenan BP, Fong L, Kelley RK (2019). Immunotherapy in hepatocellular carcinoma: the complex interface between inflammation, fibrosis, and the immune response. J Immunother Cancer.

[11] Tian Z, Hou X, Liu W, Han Z, Wei L (2019). Macrophages and hepatocellular carcinoma. Cell Biosci.

[12] Cheng Y, Ma XL, Wei YQ, Wei XW (2019). Potential roles and targeted therapy of the CXCLs/CXCR2 axis in cancer and inflammatory diseases. Biochim Biophys Acta Rev Cancer.

[13] Hao Q, Vadgama JV, Wang P (2020). CCL2/CCR2 signaling in cancer pathogenesis. Cell Commun Signal.

[14] Do HTT, Lee CH, Cho J (2020). Chemokines and their receptors: multifaceted roles in cancer progression and potential value as cancer prognostic markers. Cancers.

[15] Xue D, Zheng Y, Wen J, Han J, Tuo H, Liu Y, Peng Y (2021). Role of chemokines in hepatocellular carcinoma (Review). Oncol Rep.

[16] Li YM, Liu ZY, Wang JC, Yu JM, Li ZC, Yang HJ, Tang J, Chen ZN (2019). Receptor-interacting protein kinase 3 deficiency recruits myeloid-derived suppressor cells to hepatocellular carcinoma through the chemokine (C-X-C Motif) ligand 1-chemokine (C-X-C Motif) receptor 2 axis. Hepatology.

[17] Nywening TM, Belt BA, Cullinan DR, Panni RZ, Han BJ, Sanford DE, Jacobs RC, Ye J, Patel AA, Gillanders WE (2018). Targeting both tumour-associated CXCR2(+) neutrophils and CCR2(+) macrophages disrupts myeloid recruitment and improves chemotherapeutic responses in pancreatic ductal adenocarcinoma. Gut.

[18] Sano M, Ijichi H, Takahashi R, Miyabayashi K, Fujiwara H, Yamada T, Kato H, Nakatsuka T, Tanaka Y, Tateishi K (2019). Blocking CXCLs-CXCR2 axis in tumor-stromal interactions contributes to survival in a mouse model of pancreatic ductal adenocarcinoma through reduced cell invasion/migration and a shift of immune-inflammatory microenvironment. Oncogenesis.

[19] Li X, Yao W, Yuan Y, Chen P, Li B, Li J, Chu R, Song H, Xie D, Jiang X (2017). Targeting of tumour-infiltrating macrophages via CCL2/CCR2 signalling as a therapeutic strategy against hepatocellular carcinoma. Gut.

[20] Li T, Fu J, Zeng Z, Cohen D, Li J, Chen Q, Li B, Liu XS (2020). TIMER2.0 for analysis of tumor-infiltrating immune cells. Nucleic Acids Res.

[21] Chandrashekar DS, Bashel B, Balasubramanya SAH, Creighton CJ, Ponce-Rodriguez I, Chakravarthi B, Varambally S (2017). UALCAN: A portal for facilitating tumor subgroup gene expression and survival analyses. Neoplasia.

[22] Goldman MJ, Craft B, Hastie M, Repecka K, McDade F, Kamath A, Banerjee A, Luo Y, Rogers D, Brooks AN (2020). Visualizing and interpreting cancer genomics data via the Xena platform. Nat Biotechnol.

[23] Park JH, McMillan DC, Powell AG, Richards CH, Horgan PG, Edwards J, Roxburgh CS (2015). Evaluation of a tumor microenvironment-based prognostic score in primary operable colorectal cancer. Clin Cancer Res.

[24] Gao L, Lv G, Li R, Liu WT, Zong C, Ye F, Li XY, Yang X, Jiang JH, Hou XJ (2019). Glycochenodeoxycholate promotes hepatocellular carcinoma invasion and migration by AMPK/mTOR dependent autophagy activation. Cancer Lett.

[25] Stadtmann A, Zarbock A (2012). CXCR2: from bench to bedside. Front Immunol.

[26] Ye H, Zhou Q, Zheng S, Li G, Lin Q, Wei L, Fu Z, Zhang B, Liu Y, Li Z (2018). Tumor-associated macrophages promote progression and the Warburg effect via CCL18/NF-kB/VCAM-1 pathway in pancreatic ductal adenocarcinoma. Cell Death Dis.

[27] Bruix J, Sherman M (2011). American association for the study of liver D: management of hepatocellular carcinoma: an update. Hepatology.

[28] Lanza E, Donadon M, Poretti D, Pedicini V, Tramarin M, Roncalli M, Rhee H, Park YN, Torzilli G (2016). Transarterial therapies for hepatocellular carcinoma. Liver Cancer.

[29] Lo CM, Ngan H, Tso WK, Liu CL, Lam CM, Poon RT, Fan ST, Wong J (2002). Randomized controlled trial of transarterial lipiodol chemoembolization for unresectable hepatocellular carcinoma. Hepatology.

[30] Bharat A, Brown DB, Crippin JS, Gould JE, Lowell JA, Shenoy S, Desai NM, Chapman WC (2006). Pre-liver transplantation locoregional adjuvant therapy for hepatocellular carcinoma as a strategy to improve longterm survival. J Am Coll Surg.

[31] He S, Fan X, Ma H, Xiaerfuhazi H, Rehato A, Feng J, Shi X, He F (2019). Effect of prophylactic TACE on 5-year survival of patients with hepatocellular carcinoma after hepatectomy. Oncol Lett.

[32] Del Campo JA, Gallego P, Grande L (2018). Role of inflammatory response in liver diseases: therapeutic strategies. World J Hepatol.

[33] Tacke F (2017). Targeting hepatic macrophages to treat liver diseases. J Hepatol.

[34] Moeini P, Niedzwiedzka-Rystwej P (2021). Tumor-associated macrophages: combination of therapies, the approach to improve cancer treatment. Int J Mol Sci.

[35] Liu K, Wang FS, Xu R (2021). Neutrophils in liver diseases: pathogenesis and therapeutic targets. Cell Mol Immunol.

[36] van der Heide D, Weiskirchen R, Bansal R (2019). Therapeutic targeting of hepatic macrophages for the treatment of liver diseases. Front Immunol.

[37] Tolle F, Umansky V, Utikal J, Kreis S, Brechard S (2021). Neutrophils in tumorigenesis: missing targets for successful next generation cancer therapies?. Int J Mol Sci.

[38] Waldman AD, Fritz JM, Lenardo MJ (2020). A guide to cancer immunotherapy: from T cell basic science to clinical practice. Nat Rev Immunol.

[39] Billan S, Kaidar-Person O, Gil Z (2020). Treatment after progression in the era of immunotherapy. Lancet Oncol.

[40] Zongyi Y, Xiaowu L (2020). Immunotherapy for hepatocellular carcinoma. Cancer Lett.

[41] Anderson NR, Minutolo NG, Gill S, Klichinsky M (2021). Macrophage-based approaches for cancer immunotherapy. Can Res.

[42] Wang H, Calvisi DF, Chen X (2021). Organoids for the study of liver cancer. Semin Liver Dis.

[43] Xu ZT, Ding H, Fu TT, Zhu YL, Wang WP (2019). A nude mouse model of orthotopic liver transplantation of human hepatocellular carcinoma HCCLM3 cell xenografts and the use of imaging to evaluate tumor progression. Med Sci Monit.

[44] Heindryckx F, Colle I, Van Vlierberghe H (2009). Experimental mouse models for hepatocellular carcinoma research. Int J Exp Pathol.

