# Supplementary material for: Targeted blocking of CCR2 and CXCR2 improves the efficacy of transarterial chemoembolization of hepatocarcinoma
Source: Cancer Cell Int. 2022 Nov 19;22:362. doi: 10.1186/s12935-022-02771-z (PMC9675208; doi:10.1186/s12935-022-02771-z)
Supplement: Supplementary file 1 — Additional file 1: Figure S1. Neutrophil and macrophage infiltration and CXCL8expression are associated with poor prognosis. （A-F）TIMER2.0 is a comprehensive database for systematical analysis of the abundances of different immune infiltrates across 32 cancer types from TCGA. We explored the correlation between six immune subsets (CD8+ T cells, CD4+ T cells, B cells, Neutrphils, Macrophages and Dendritic cells) and survival rate of HCC patients. The Kaplan- Meier plots were drawn with TIMER2.0 for immune infiltrates and HCC to visualize the survival differences. Immune cells infiltrate levels are divided into low- and high- level groups by the split percentage of patients 50%. The hazard ratio (HR) for the Cox model and the log-rank p-value for Kaplan–Meier curves are shown. (G) The expression levels of CCL2, CCR2, CXCL8 and CXCR2 in HCC tissues were explored with RNA sequencing data from the TCGA liver cancer dataset, and analyzed by the UALCAN web server (http://ualcan.path.uab.edu/index.html). Kaplan-Meier OS curves are shown according to low and high expression of CCL2, CCR2, CXCL8, and CXCR2. p < 0.05 was considered as statistically significant. Figure S2. Correlation of CCL2/CCR2 and CXCLs/CXCR2 genes’ expression with immune infiltration level in HCC. The correlation between genes expression of CCL2/CCR2 and CXCLs/CXCR2 and immune infiltrates were evaluated via the TIMER 2.0 web server in the TCGA liver cancer cohort. TIMER2.0 is freely available at http://timer.cistrome.org. Scatter plots show the relationship between gene (CCL2, CCR2, CXCL1, CXCL8, and CXCR2) expression level and six infiltrating immune cells (CD8+ T cells, CD4+ T cells, B cells, dendritic cells, neutrophils, and macrophages) estimation value by TIMER algorithm based on RNA-Seq expression profiles data. Figure S3. Compare CCL2/CCR2 and CXCLs/CXCR2 genes’ expression of tumor and adjacent normal tissues in HCC. The UCSC Xena browser (https://xenabrowser.net) was utilized to obtain the correspo [file 12935_2022_2771_MOESM1_ESM.docx]

**Supplemental Information**

# Targeted blocking of CCR2 and CXCR2 improves the efficacy of transarterial chemoembolization of hepatocarcinoma

Zhiqiang Tian1, 4, *, Xiaojuan Hou1, 2, *, Wenting Liu1, 2, *, Changchun Shao1, 2, Lu Gao1,

2, Jinghua Jiang1, 2, Li Zhang3, #, Zhipeng Han1, 2, #, Lixin Wei1, 2, #

# Supplemental Figures and Tables


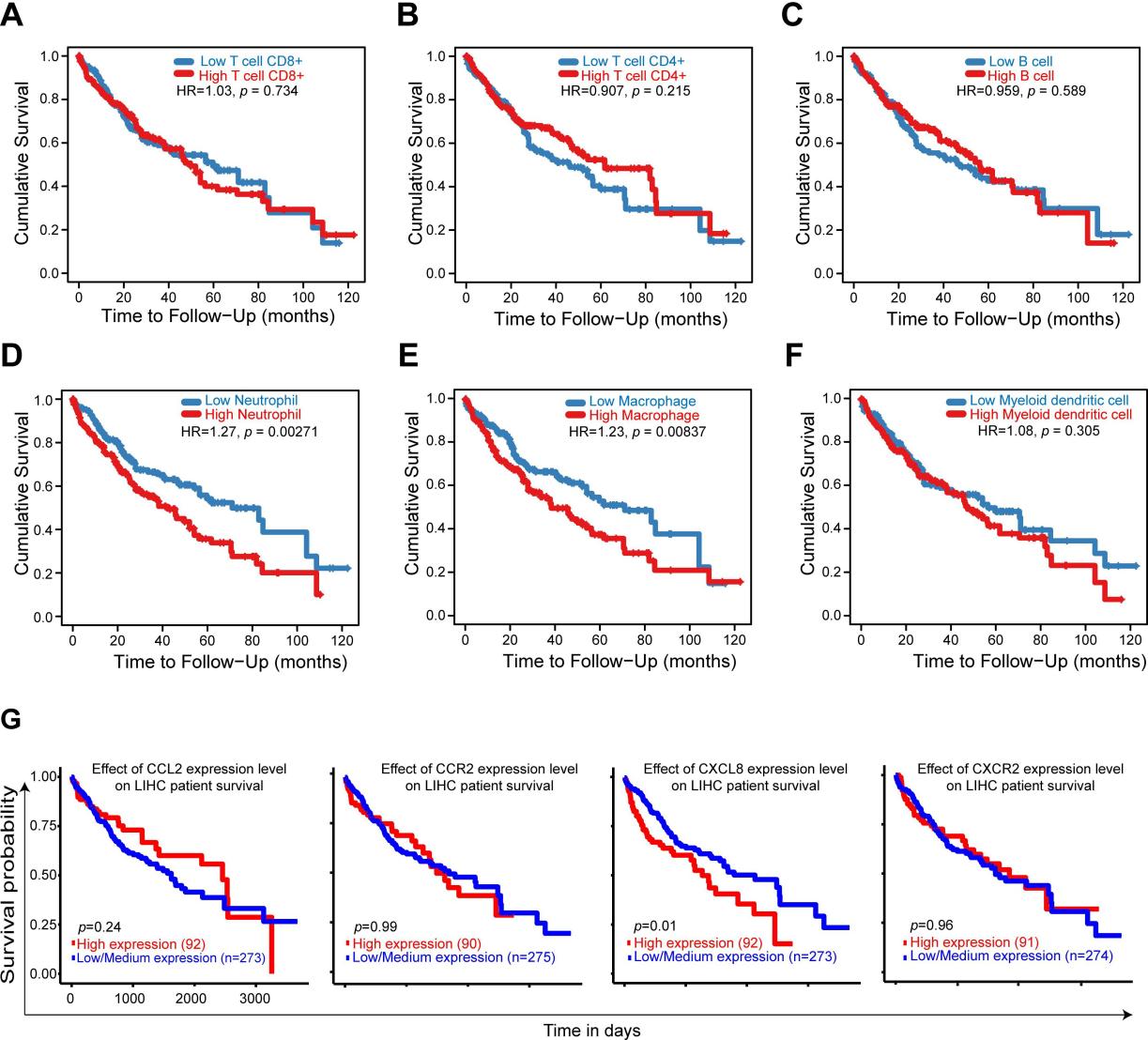


**Supplementary Figure 1: Neutrophil and macrophage infiltration and CXCL8**

**expression are associated with poor prognosis.** （A-F）TIMER2.0 is a comprehensive database for systematical analysis of the abundances of different immune infiltrates across 32 cancer types from TCGA. We explored the correlation between six immune subsets (CD8+ T cells, CD4+ T cells, B cells, Neutrphils, Macrophages and Dendritic cells) and survival rate of HCC patients. The Kaplan- Meier plots were drawn with TIMER2.0 for immune infiltrates and HCC to visualize the survival differences. Immune cells infiltrate levels are divided into low- and high- level groups by the split percentage of patients 50%. The hazard ratio (HR) for the Cox model and the log-rank p-value for Kaplan–Meier curves are shown. (G) The expression levels of CCL2, CCR2, CXCL8 and CXCR2 in HCC tissues were explored with RNA sequencing data from the TCGA liver cancer dataset, and analyzed by the UALCAN web server (http://ualcan.path.uab.edu/index.html). Kaplan-Meier OS curves are shown according to low and high expression of CCL2, CCR2, CXCL8, and CXCR2. p < 0.05 was considered as statistically significant.


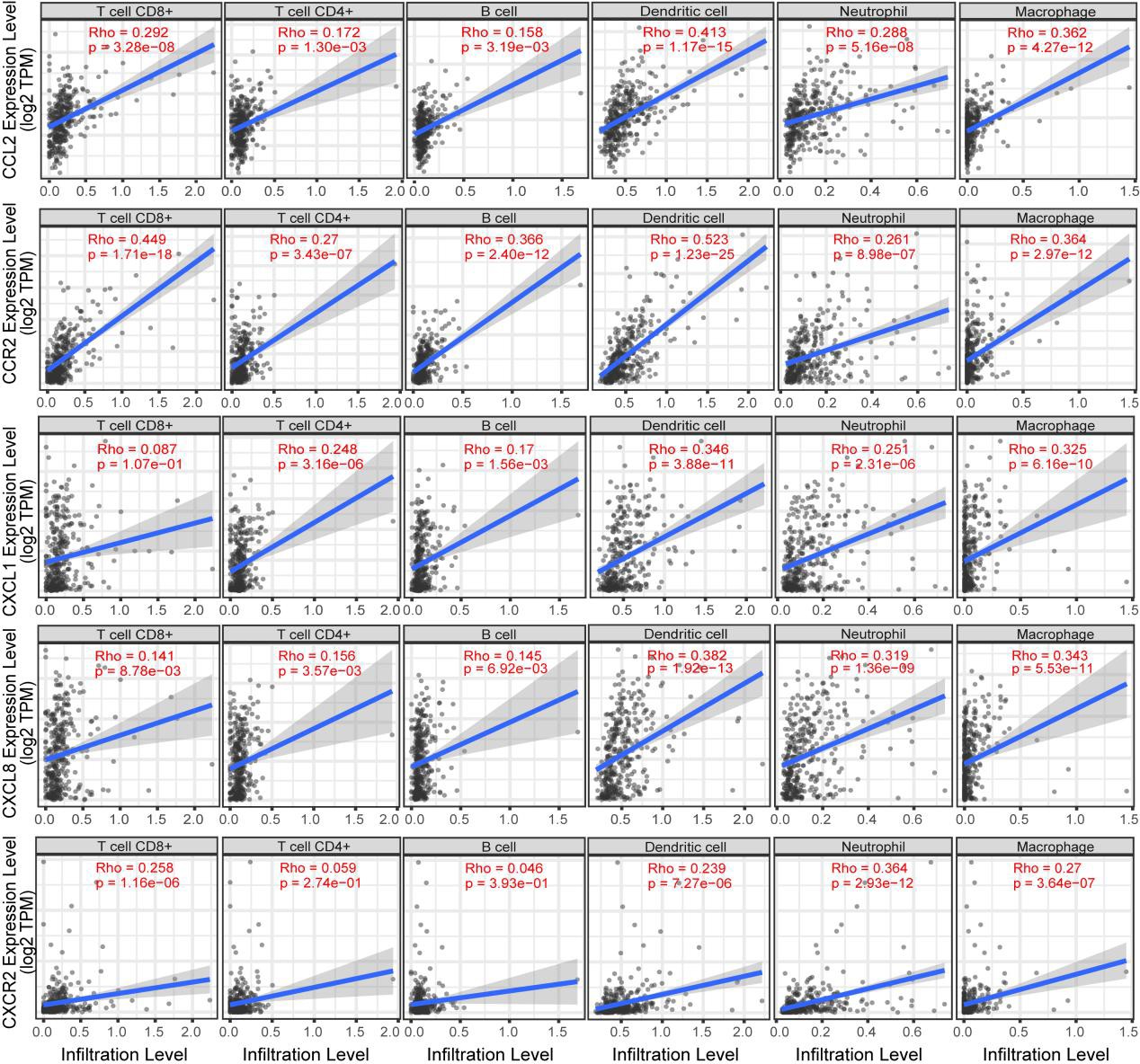


**Supplementary Figure 2: Correlation of CCL2/CCR2 and CXCLs/CXCR2 genes’ expression with immune infiltration level in HCC.** The correlation between genes expression of CCL2/CCR2 and CXCLs/CXCR2 and immune infiltrates were evaluated via the TIMER 2.0 web server in the TCGA liver cancer cohort. TIMER2.0 is freely available at [http://timer.cistrome.org.](http://timer.cistrome.org/) Scatter plots show the relationship between gene (CCL2, CCR2, CXCL1, CXCL8, and CXCR2) expression level and six infiltrating immune cells (CD8+ T cells, CD4+ T cells, B cells, dendritic cells, neutrophils, and macrophages) estimation value by TIMER algorithm based on RNA-Seq expression profiles data.


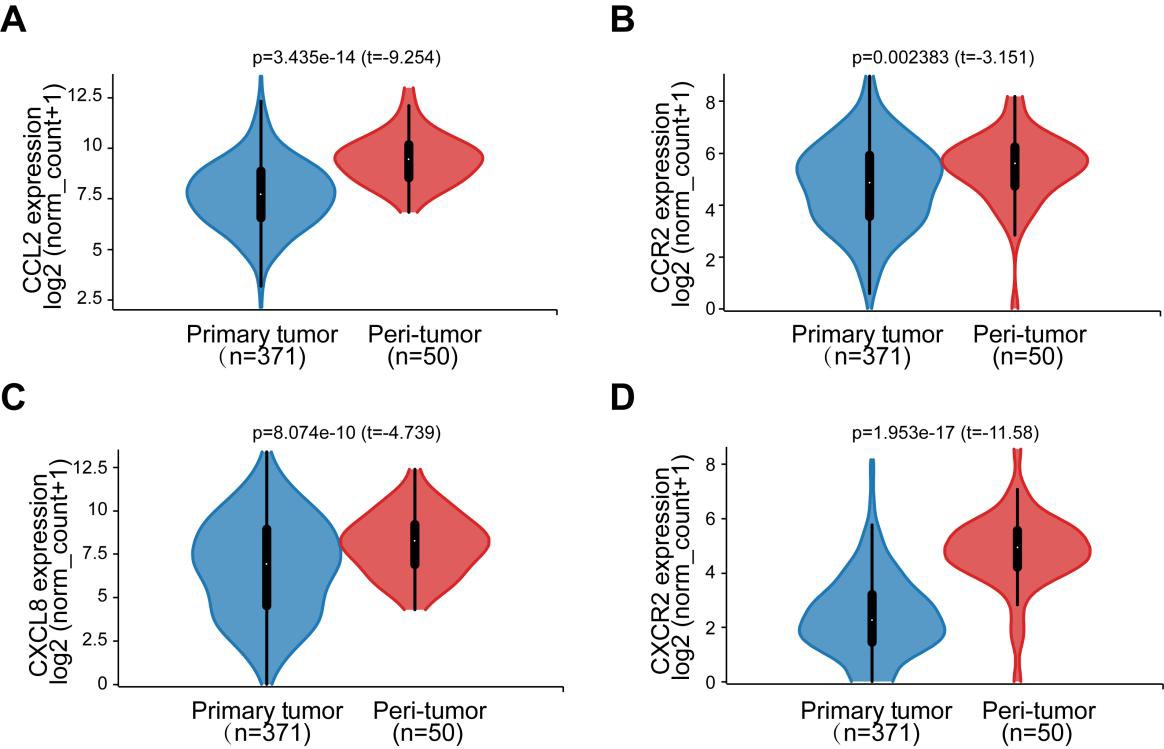


**Supplementary Figure 3: Compare CCL2/CCR2 and CXCLs/CXCR2 genes’ expression of tumor and adjacent normal tissues in HCC.** The UCSC Xena browser (https://xenabrowser.net) was utilized to obtain the corresponding violin plots of the mRNA expression levels of CCL2/CCR2 and CXCL8/CXCR2 between tumor and adjacent normal tissues of TCGA liver cancer database. (A) Different expression of CCL2 between HCC and adjacent normal tissues (*p* = 3.435e-14). (B) Different expression of CCR2 between HCC and adjacent normal tissues (*p* = 0.002). (C) Different expression of CXCL8 between HCC and adjacent normal tissues (*p* = 0.000008). (D) Different expression of CXCR2 between HCC and adjacent normal tissues (*p* = 1.953e-17). Primary tumor (n=371), adjacent normal tissue(n=50). Welch's t test, *p* value <0.05.


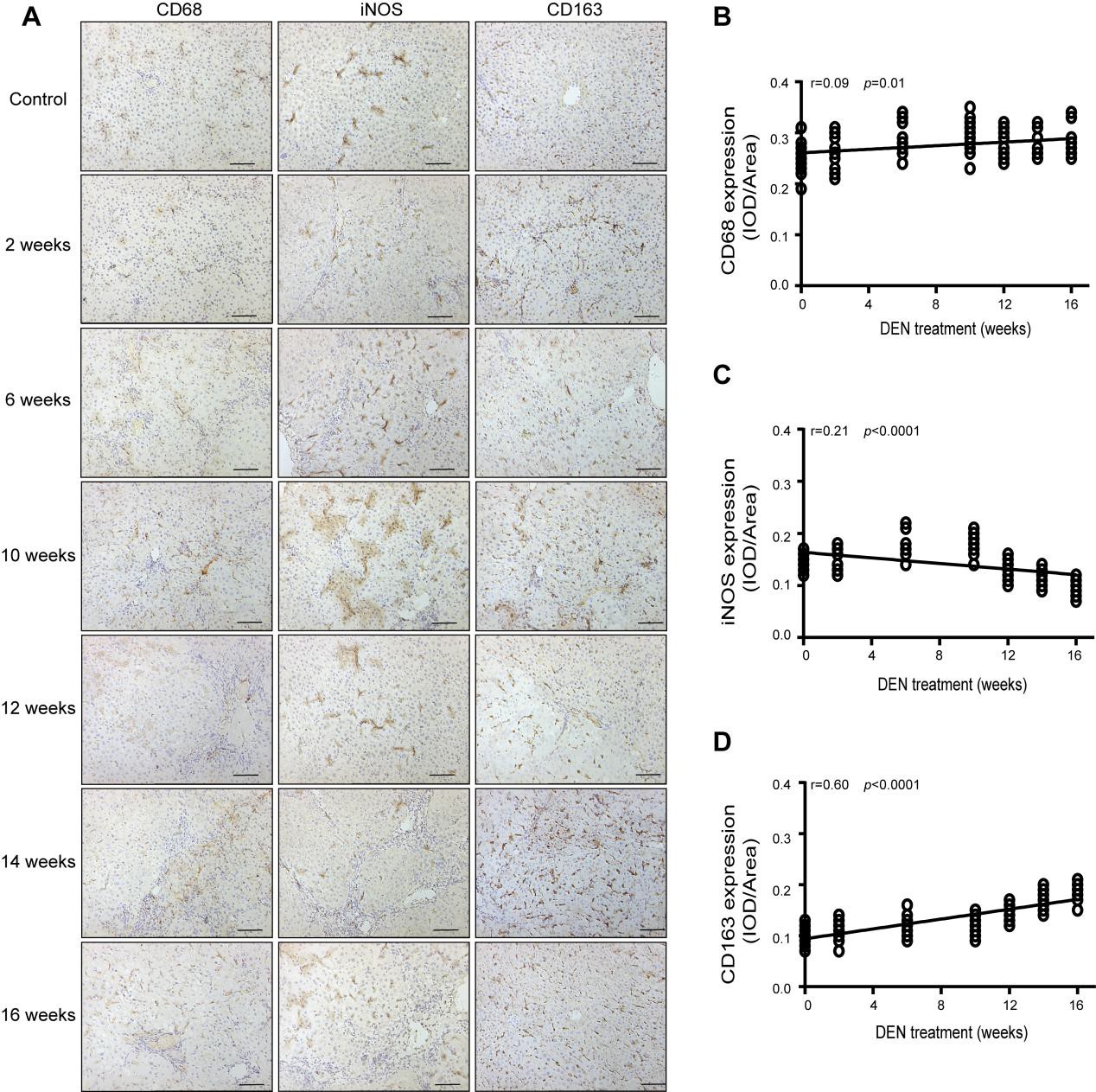


**Supplementary Figure 4: Macrophage phenotypic switched correlated with hepatocarcinogenesis progression in primary rat HCC model.** Sprague Dawley rats were treated with DEN. The rats were sacrificed to observe the development of HCC. To understand the phenotypes of infiltrated macrophages in rat hepatocarcinogenesis progression, the expression of CD68 (a general macrophage marker), iNOS (a surface marker for M1 macrophage phenotype), and CD163 (a surface marker for M2 macrophage phenotype) were detected by immunohistochemical. The stained sections were evaluated by IOD, and then the correlation of the gene expression level and DEN treatment times was analyzed by the

Pearson’s correlation. Pearson’s correlation analysis provided correlation coefficient

(*r*) and *p*-value. (A) Representative immunohistochemical images of CD68, iNOS, and CD163 staining in rat liver tissue of the indicated groups were shown. (B) Pearson's correlation analysis between CD68 and DEN treatment times (r = 0.09, p = 0.01). (C) Pearson's correlation analysis between iNOS and DEN treatment times (r = 0.21, p < 0.0001). (D) Pearson's correlation analysis between CD163 and DEN treatment times (r = 0.60, p < 0.0001).


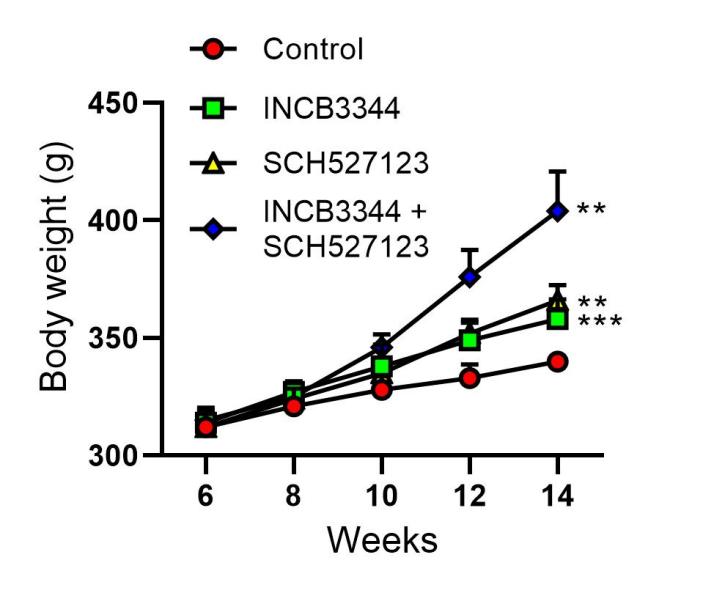


**Supplementary Figure 5:** **Intraperitoneal administration of CCR2 and CXCR2 antagonists prevents hepatocarcinogenesis in the primary HCC model.** After 8 weeks of diethylnitrosamine (DEN) treatment, Sprague Dawley rats were intraperitoneally injected with the CCR2 antagonist INCB3344 (60 μg/g body weight) in 200 μl saline, the CXCR2 antagonist SCH527123 (10 μg/g body weight) in 200 μl saline, INCB3344 (60 μg/g) + SCH527123 (10 μg/g) in 200 μl saline, or 200 μl of blank saline. Injections were continued twice every week for 6 weeks. After 14 weeks, the rats were sacrificed to observe the development of HCC. The body weight of the rats was recorded. Data are presented as mean ± SD. ***p* < 0.01, ****p* < 0.001.

**
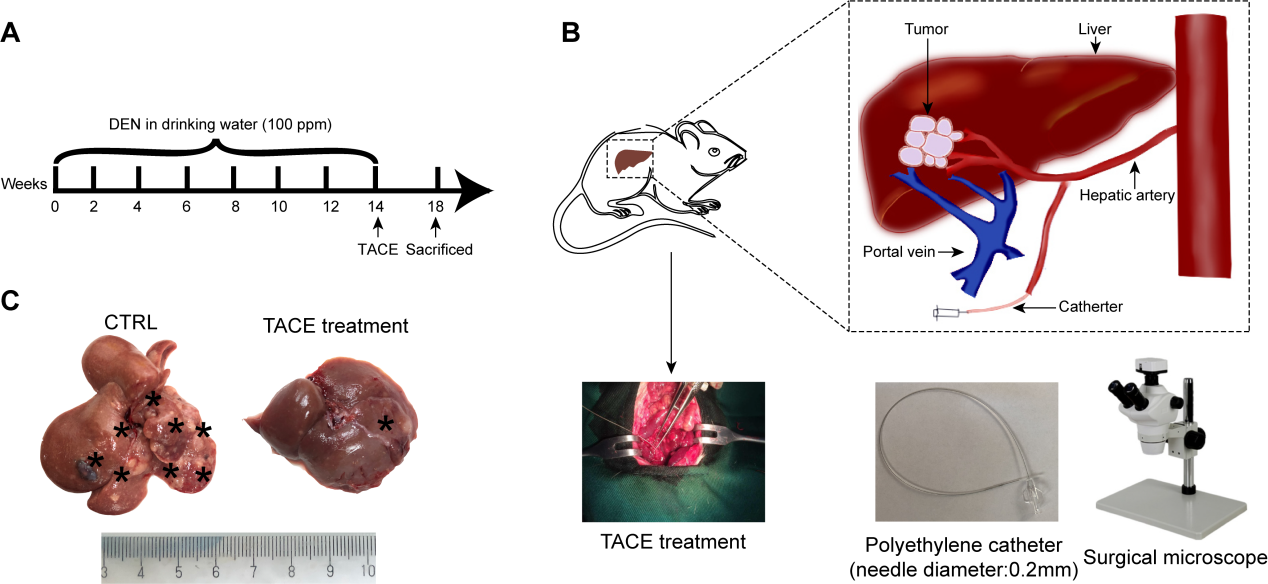
**

**Supplementary Figure 6: TACE in primary rat HCC model.** (A) Sprague Dawley rats with DEN-induced HCC were used to simulate human liver cancer. TACE procedure was performed in rat after 14 weeks DEN treatment. (B) TACE procedure was modified in the primary rat HCC model. Briefly, a polyethylene catheter (PE-10) with a 0.6-mm outside diameter connected to one end of a needle (inner diameter: 0.2mm) was used for catheterization under laparotomy. After exposure of the liver tumor, the needle was inserted retrogradely into the gastroduodenal artery by using a binocular operative microscope. The mentioned agents were injected through the catheter to the hepatic artery. The hepatic artery was then ligated. (C) Representative images of rat livers of the control and TACE treatment group, the typical tumor nodes were shown by the asterisks.


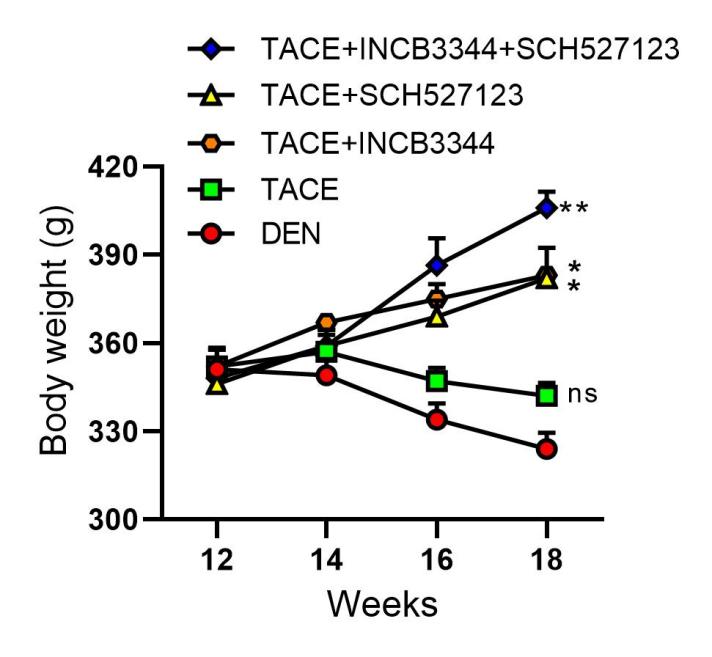


**Supplementary Figure 7: Blockade of CCL2/CCR2 and CXCL1/CXCR2 enhances the antitumor effect of TACE in the HCC model.** Sprague Dawley rats were treated with diethylnitrosamine (DEN) for 14 weeks, then subjected to the modified TACE procedure. The chemotherapeutic drug cisplatin (3 mg/kg body weight) was administered to the TACE-treated rats with or without the CCR2 antagonist INCB3344 (60 μg/g body weight) and the CXCR2 antagonist SCH527123 (10 μg/g body weight). Control animals received the TACE procedure and administered 0.5ml normal saline. The body weight of the rats was recorded. Data are presented as mean ± SD. ns, not statistically significant, **p* < 0.05, ***p* < 0.01.

Supplementary Table 1. HCC-associated chemokine families and their functions in immune infiltrates

| HCC‑associated chemokine receptors | HCC‑associated chemokines | Functions in immune infiltrates |
| --- | --- | --- |
| CCR1 | CCL3, CCL5, CCL14, CCL23 | T cells, Monocytes |
| CCR2 | CCL2 | Macrophages |
| CCR3 | CCL24 | Eosinophils |
| CCR4 | CCL17, CCL22 | T cells, Monocytes |
| CCR5 | CCL4, CCL5 | Macrophages, T cells |
| CCR6 | CCL20 | Dendritic cells, T cells CD4+ |
| CCR7 | CCL19 | T cells |
| CCR8 | CCL1 | T cells |
| CCR9 | CCL25 | T cells |
| CCR10 | CCL27, CCL28 | B cells |
| CXCR1 | CXCL6, CXCL8 | Neutrophils |
| CXCR2 | CXCL1, CXCL2, CXCL3, CXCL5, CXCL8 | Neutrophils |
| CXCR3 | CXCL9, CXCL10, CXCL11 | T cells |
| CXCR4, CXCR7 | CXCL12 | B cells |
| CXCR5 | CXCL13 | B cells |
| CXCR6 | CXCL16 | T cells |
| XCR1 | XCL1, XCL2 | Tregs |
| CX3CR1 | CX3CL1 | Natural killer cells |

Supplementary Table 2. The relationship between HCC-associated chemokine expression and the infiltrate estimation value for CD8+ T cells in the TCGA LIHC dataset

| HCC‑associated chemokines | infiltrates estimation value | rho | *p* | adjustment *p* |
| --- | --- | --- | --- | --- |
| CCR1 | T cell CD8+_TIMER | **0.494** | **1.20E-22** | **1.60E-21** |
| CCL3 | T cell CD8+_TIMER | **0.419** | **4.33E-16** | **4.12E-15** |
| CCL5 | T cell CD8+_TIMER | **0.578** | **4.44E-32** | **1.54E-31** |
| CCL14 | T cell CD8+_TIMER | **0.048** | **0.379** | **0.569** |
| CCL23 | T cell CD8+_TIMER | **0.250** | **2.59E-06** | **1.30E-05** |
| CCR2 | T cell CD8+_TIMER | **0.449** | **1.71E-18** | **6.69E-18** |
| CCL2 | T cell CD8+_TIMER | **0.292** | **3.28E-08** | **2.43E-07** |
| CCR3 | T cell CD8+_TIMER | **0.152** | **0.005** | **0.013** |
| CCL24 | T cell CD8+_TIMER | **0.217** | **4.74E-05** | **0.001** |
| CCR4 | T cell CD8+_TIMER | **0.400** | **1.09E-14** | **5.01E-14** |
| CCL17 | T cell CD8+_TIMER | **0.212** | **7.14E-05** | **0.0003** |
| CCL22 | T cell CD8+_TIMER | **0.268** | **4.33E-07** | **1.77E-06** |
| CCR5 | T cell CD8+_TIMER | **0.542** | **8.80E-28** | **3.45E-27** |
| CCL4 | T cell CD8+_TIMER | **0.589** | **1.36E-33** | **8.91E-33** |
| CCR6 | T cell CD8+_TIMER | 0.026 | 0.624 | 0.710 |
| CCL20 | T cell CD8+_TIMER | 0.001 | 0.988 | 0.995 |
| CCR7 | T cell CD8+_TIMER | **0.403** | **7.09E-15** | **2.55E-14** |
| CCL19 | T cell CD8+_TIMER | **0.255** | **1.58E-06** | **3.89E-06** |
| CCR8 | T cell CD8+_TIMER | **0.388** | **7.15E-14** | **3.67E-13** |
| CCL1 | T cell CD8+_TIMER | 0.034 | 0.532 | 0.665 |
| CCR9 | T cell CD8+_TIMER | **0.196** | **0.0003** | **0.001** |
| CCL25 | T cell CD8+_TIMER | 0.065 | 0.228 | 0.324 |
| CCR10 | T cell CD8+_TIMER | 0.091 | 0.092 | 0.226 |
| CCL27 | T cell CD8+_TIMER | 0.038 | 0.479 | 0.834 |
| CCL28 | T cell CD8+_TIMER | **0.168** | **0.002** | **0.016** |
| CXCR1 | T cell CD8+_TIMER | **0.145** | **0.007** | **0.035** |
| CXCL6 | T cell CD8+_TIMER | 0.091 | 0.090 | 0.215 |
| CXCR2 | T cell CD8+_TIMER | **0.258** | **1.16E-06** | **2.32E-05** |
| CXCL1 | T cell CD8+_TIMER | 0.087 | 0.107 | 0.247 |
| CXCL2 | T cell CD8+_TIMER | 0.083 | 0.122 | 0.339 |
| CXCL3 | T cell CD8+_TIMER | **0.162** | **0.003** | **0.019** |
| CXCL5 | T cell CD8+_TIMER | 0.038 | 0.484 | 0.686 |
| CXCL8 | T cell CD8+_TIMER | **0.141** | **0.009** | **0.032** |
| CXCR3 | T cell CD8+_TIMER | **0.446** | **2.88E-18** | **7.16E-18** |
| CXCL9 | T cell CD8+_TIMER | **0.505** | **1.09E-23** | **3.90E-23** |
| CXCL10 | T cell CD8+_TIMER | **0.466** | **5.79E-20** | **2.27E-19** |
| CXCL11 | T cell CD8+_TIMER | **0.496** | **8.94E-23** | **4.70E-22** |
| CXCR4 | T cell CD8+_TIMER | **0.416** | **7.53E-16** | **4.18E-15** |
| CXCR7 | T cell CD8+_TIMER | **0.113** | **0.036** | **0.091** |
| CXCL12 | T cell CD8+_TIMER | **0.195** | **0.0003** | **0.001** |

| CXCR5 | T cell CD8+_TIMER | **0.286** | **6.18E-08** | **1.52E-07** |
| --- | --- | --- | --- | --- |
| CXCL13 | T cell CD8+_TIMER | **0.258** | **1.15E-06** | **2.19E-06** |
| CXCR6 | T cell CD8+_TIMER | **0.572** | **2.01E-31** | **6.70E-31** |
| CXCL16 | T cell CD8+_TIMER | **0.107** | **0.047** | **0.088** |
| XCR1 | T cell CD8+_TIMER | **0.314** | **2.48E-09** | **1.13E-08** |
| XCL1 | T cell CD8+_TIMER | **0.294** | **2.66E-08** | **7.19E-08** |
| XCL2 | T cell CD8+_TIMER | **0.395** | **2.38E-14** | **5.74E-14** |
| CX3CR1 | T cell CD8+_TIMER | **0.282** | **1.00E-07** | **9.79E-07** |
| CX3CL1 | T cell CD8+_TIMER | **0.215** | **5.67E-05** | **0.0003** |

The bold text means positive correlation (Spearman’s *p* < 0.05, *p* > 0), the others mean not

significant (Spearman’s *p* > 0.05).

Supplementary Table 3. The relationship between HCC-associated chemokines expression and the infiltrate estimation value for CD4+ T cells in the TCGA LIHC dataset

| HCC-associated chemokines | infiltrates estimation value | rho | *p* | adjustment *p* |
| --- | --- | --- | --- | --- |
| CCR1 | T cell CD4+_TIMER | 0.086 | 0.111 | 0.171 |
| CCL3 | T cell CD4+_TIMER | -0.105 | 0.052 | 0.122 |
| CCL5 | T cell CD4+_TIMER | **0.131** | **0.015** | **0.029** |
| CCL14 | T cell CD4+_TIMER | -0.046 | 0.398 | 0.559 |
| CCL23 | T cell CD4+_TIMER | 0.065 | 0.230 | 0.399 |
| CCR2 | T cell CD4+_TIMER | **0.270** | **3.43E-07** | **1.52E-06** |
| CCL2 | T cell CD4+_TIMER | **0.172** | **0.001** | **0.006** |
| CCR3 | T cell CD4+_TIMER | 0.082 | 0.127 | 0.281 |
| CCL24 | T cell CD4+_TIMER | 0.048 | 0.374 | 0.591 |
| CCR4 | T cell CD4+_TIMER | **0.234** | **1.08E-05** | **3.93E-05** |
| CCL17 | T cell CD4+_TIMER | **0.270** | **3.42E-07** | **3.77E-06** |
| CCL22 | T cell CD4+_TIMER | **0.371** | **1.09E-12** | **1.81E-11** |
| CCR5 | T cell CD4+_TIMER | **0.295** | **2.38E-08** | **1.02E-07** |
| CCL4 | T cell CD4+_TIMER | -0.044 | 0.418 | 0.494 |
| CCR6 | T cell CD4+_TIMER | **0.479** | **3.42E-21** | **7.19E-20** |
| CCL20 | T cell CD4+_TIMER | **0.284** | **8.26E-08** | **2.17E-06** |
| CCR7 | T cell CD4+_TIMER | **0.256** | **1.43E-06** | **6.95E-06** |
| CCL19 | T cell CD4+_TIMER | **0.256** | **1.41E-06** | **8.74E-06** |
| CCR8 | T cell CD4+_TIMER | **0.234** | **1.09E-05** | **4.42E-05** |
| CCL1 | T cell CD4+_TIMER | **0.234** | **1.09E-05** | **4.42E-05** |
| CCR9 | T cell CD4+_TIMER | **0.185** | **0.001** | **0.003** |
| CCL25 | T cell CD4+_TIMER | 0.079 | 0.145 | 0.294 |
| CCR10 | T cell CD4+_TIMER | **0.287** | **5.78E-08** | **1.14E-06** |
| CCL27 | T cell CD4+_TIMER | 0.044 | 0.418 | 0.670 |
| CCL28 | T cell CD4+_TIMER | **0.227** | **2.04E-05** | **0.0003** |
| CXCR1 | T cell CD4+_TIMER | 0.042 | 0.436 | 0.698 |
| CXCL6 | T cell CD4+_TIMER | **0.165** | **0.002** | **0.018** |
| CXCR2 | T cell CD4+_TIMER | 0.059 | 0.274 | 0.471 |
| CXCL1 | T cell CD4+_TIMER | **0.248** | **3.16E-06** | **7.38E-05** |
| CXCL2 | T cell CD4+_TIMER | -0.028 | 0.607 | 0.809 |

| CXCL3 | T cell CD4+_TIMER | **0.246** | **3.82E-06** | **6.17E-05** |
| --- | --- | --- | --- | --- |
| CXCL5 | T cell CD4+_TIMER | **0.206** | **0.0001** | **0.001** |
| CXCL8 | T cell CD4+_TIMER | **0.156** | **0.004** | **0.016** |
| CXCR3 | T cell CD4+_TIMER | **0.279** | **1.45E-07** | **6.96E-07** |
| CXCL9 | T cell CD4+_TIMER | 0.024 | 0.652 | 0.721 |
| CXCL10 | T cell CD4+_TIMER | -0.044 | 0.415 | 0.501 |
| CXCL11 | T cell CD4+_TIMER | -0.057 | 0.29 | 0.379 |
| CXCR4 | T cell CD4+_TIMER | **0.328** | **4.20E-10** | **4.34E-09** |
| CXCR7 | T cell CD4+_TIMER | 0.062 | 0.248 | 0.462 |
| CXCL12 | T cell CD4+_TIMER | **0.282** | **1.00E-07** | **9.74E-07** |
| CXCR5 | T cell CD4+_TIMER | **0.347** | **3.49E-11** | **3.54E-10** |
| CXCL13 | T cell CD4+_TIMER | 0.072 | 0.179 | 0.274 |
| CXCR6 | T cell CD4+_TIMER | **0.19** | **0.0004** | **0.001** |
| CXCL16 | T cell CD4+_TIMER | **0.282** | **9.62E-08** | **1.29E-06** |
| XCR1 | T cell CD4+_TIMER | **0.156** | **0.004** | **0.012** |
| XCL1 | T cell CD4+_TIMER | **0.157** | **0.003** | **0.014** |
| XCL2 | T cell CD4+_TIMER | 0.091 | 0.09 | 0.156 |
| CX3CR1 | T cell CD4+_TIMER | **0.22** | **3.77E-05** | **0.0002** |
| CX3CL1 | T cell CD4+_TIMER | **0.223** | **2.80E-05** | **0.0003** |

The bold text means positive correlation (Spearman’s *p* < 0.05, *p* > 0), the others mean not significant (Spearman’s *p* > 0.05).

Supplementary Table 4. The relationship between HCC-associated chemokine expression and the infiltrate estimation value for B cells in the TCGA LIHC dataset

| HCC‑associated chemokines | infiltrates estimation value | rho | *p* | adjustment *p* |
| --- | --- | --- | --- | --- |
| CCR1 | B cell_TIMER | **0.241** | **6.25E-06** | **3.23E-05** |
| CCL3 | B cell_TIMER | **0.169** | **0.002** | **0.007** |
| CCL5 | B cell_TIMER | **0.345** | **4.47E-11** | **2.48E-10** |
| CCL14 | B cell_TIMER | -0.291# | 3.89E-08# | 3.53E-07# |
| CCL23 | B cell_TIMER | 0.002 | 0.969 | 0.988 |
| CCR2 | B cell_TIMER | **0.366** | **2.40E-12** | **1.24E-11** |
| CCL2 | B cell_TIMER | **0.158** | **0.003** | **0.012** |
| CCR3 | B cell_TIMER | **0.171** | **0.001** | **0.009** |
| CCL24 | B cell_TIMER | **0.127** | **0.019** | **0.079** |
| CCR4 | B cell_TIMER | **0.305** | **7.64E-09** | **3.40E-08** |
| CCL17 | B cell_TIMER | **0.192** | **0.0003** | **0.001** |
| CCL22 | B cell_TIMER | **0.294** | **2.73E-08** | **1.49E-07** |
| CCR5 | B cell_TIMER | **0.462** | **1.12E-19** | **1.12E-18** |
| CCL4 | B cell_TIMER | **0.281** | **1.15E-07** | **6.66E-07** |
| CCR6 | B cell_TIMER | **0.378** | **3.69E-13** | **2.05E-12** |
| CCL20 | B cell_TIMER | **0.229** | **1.69E-05** | **0.0003** |
| CCR7 | B cell_TIMER | **0.309** | **4.32E-09** | **1.42E-08** |
| CCL19 | B cell_TIMER | **0.250** | **2.46E-06** | **6.57E-06** |
| CCR8 | B cell_TIMER | **0.337** | **1.25E-10** | **9.01E-10** |
| CCL1 | B cell_TIMER | **0.161** | **0.003** | **0.017** |
| CCR9 | B cell_TIMER | 0.080 | 0.137 | 0.234 |
| CCL25 | B cell_TIMER | 0.097 | 0.073 | 0.137 |
| CCR10 | B cell_TIMER | **0.243** | **5.01E-06** | **4.49E-05** |
| CCL27 | B cell_TIMER | 0.064 | 0.238 | 0.624 |
| CCL28 | B cell_TIMER | 0.055 | 0.313 | 0.544 |
| CXCR1 | B cell_TIMER | -0.043 | 0.422 | 0.689 |
| CXCL6 | B cell_TIMER | **0.128** | **0.017** | **0.107** |
| CXCR2 | B cell_TIMER | 0.046 | 0.393 | 0.646 |
| CXCL1 | B cell_TIMER | **0.170** | **0.002** | **0.014** |
| CXCL2 | B cell_TIMER | -0.009 | 0.861 | 0.959 |
| CXCL3 | B cell_TIMER | **0.249** | **2.97E-06** | **8.92E-05** |
| CXCL5 | B cell_TIMER | **0.161** | **0.003** | **0.025** |
| CXCL8 | B cell_TIMER | **0.145** | **0.007** | **0.031** |
| CXCR3 | B cell_TIMER | **0.423** | **2.11E-16** | **1.29E-15** |
| CXCL9 | B cell_TIMER | **0.343** | **6.10E-11** | **3.94E-10** |
| CXCL10 | B cell_TIMER | **0.250** | **2.69E-06** | **1.54E-05** |
| CXCL11 | B cell_TIMER | **0.242** | **5.71E-06** | **3.64E-05** |
| CXCR4 | B cell_TIMER | **0.478** | **4.41E-21** | **6.78E-20** |
| CXCR7 | B cell_TIMER | -0.015 | 0.777 | 0.873 |
| CXCL12 | B cell_TIMER | **0.135** | **0.012** | **0.036** |
| CXCR5 | B cell_TIMER | **0.435** | **2.30E-17** | **1.00E-16** |
| CXCL13 | B cell_TIMER | **0.252** | **2.10E-06** | **5.58E-06** |
| CXCR6 | B cell_TIMER | **0.428** | **8.97E-17** | **6.65E-16** |
| CXCL16 | B cell_TIMER | **0.348** | **2.93E-11** | **7.32E-10** |
| XCR1 | B cell_TIMER | **0.186** | **0.001** | **0.002** |
| XCL1 | B cell_TIMER | **0.260** | **9.83E-07** | **5.96E-06** |
| XCL2 | B cell_TIMER | **0.310** | **3.86E-09** | **2.03E-08** |
| CX3CR1 | B cell_TIMER | **0.190** | **0.0004** | **0.002** |
| CX3CL1 | B cell_TIMER | **0.244** | **4.40E-06** | **4.98E-05** |

The bold text means positive correlation (Spearman’s *p* < 0.05, *p* > 0), the symbol # means negative correlation (Spearman’s *p* < 0.05, *p* < 0), the others mean not significant (Spearman’s *p* > 0.05).

Supplementary Table 5. The relationship between HCC-associated chemokine expression and the infiltrate estimation value for neutrophils in the TCGA LIHC dataset

| HCC‑associated chemokines | infiltrates estimation  value | rho | *p* | adjustment *p* |
| --- | --- | --- | --- | --- |
| CCR1 | Neutrophil_TIMER | **0.376** | **5.36E-13** | **4.13E-12** |
| CCL3 | Neutrophil_TIMER | **0.233** | **1.18E-05** | **7.85E-05** |
| CCL5 | Neutrophil_TIMER | **0.239** | **7.46E-06** | **5.09E-05** |
| CCL14 | Neutrophil_TIMER | -0.198# | 0.0002# | 0.002# |
| CCL23 | Neutrophil_TIMER | 0.069 | 0.202 | 0.413 |
| CCR2 | Neutrophil_TIMER | **0.261** | **8.98E-07** | **5.65E-06** |
| CCL2 | Neutrophil_TIMER | **0.288** | **5.16E-08** | **3.85E-07** |
| CCR3 | Neutrophil_TIMER | **0.338** | **1.08E-10** | **1.07E-09** |
| CCL24 | Neutrophil_TIMER | 0.088 | 0.102 | 0.219 |
| CCR4 | Neutrophil_TIMER | **0.328** | **4.40E-10** | **3.63E-09** |
| CCL17 | Neutrophil_TIMER | **0.118** | **0.029** | **0.131** |
| CCL22 | Neutrophil_TIMER | **0.237** | **8.48E-06** | **5.96E-05** |
| CCR5 | Neutrophil_TIMER | **0.299** | **1.43E-08** | **1.07E-07** |
| CCL4 | Neutrophil_TIMER | **0.308** | **5.16E-09** | **3.62E-08** |
| CCR6 | Neutrophil_TIMER | 0.087 | 0.108 | 0.229 |
| CCL20 | Neutrophil_TIMER | **0.118** | **0.029** | **0.055** |
| CCR7 | Neutrophil_TIMER | **0.266** | **5.53E-07** | **4.89E-06** |
| CCL19 | Neutrophil_TIMER | **0.116** | **0.031** | **0.093** |
| CCR8 | Neutrophil_TIMER | **0.312** | **3.24E-09** | **2.76E-08** |
| CCL1 | Neutrophil_TIMER | **0.108** | **0.044** | **0.251** |
| CCR9 | Neutrophil_TIMER | **0.181** | **0.001** | **0.006** |
| CCL25 | Neutrophil_TIMER | -0.178# | 0.001# | 0.007# |
| CCR10 | Neutrophil_TIMER | **0.261** | **9.17E-07** | **4.38E-05** |
| CCL27 | Neutrophil_TIMER | 0.017 | 0.755 | 0.980 |
| CCL28 | Neutrophil_TIMER | **0.178** | **0.001** | **0.006** |
| CXCR1 | Neutrophil_TIMER | **0.249** | **2.91E-06** | **8.18E-06** |
| CXCL6 | Neutrophil_TIMER | **0.203** | **0.0001** | **0.001** |
| CXCR2 | Neutrophil_TIMER | **0.364** | **2.93E-12** | **1.31E-11** |
| CXCL1 | Neutrophil_TIMER | **0.251** | **2.31E-06** | **1.12E-05** |
| CXCL2 | Neutrophil_TIMER | -0.018 | 0.737 | 0.800 |
| CXCL3 | Neutrophil_TIMER | **0.293** | **2.79E-08** | **3.33E-07** |
| CXCL5 | Neutrophil_TIMER | **0.216** | **5.32E-05** | **0.0002** |
| CXCL8 | Neutrophil_TIMER | **0.319** | **1.36E-09** | **5.89E-09** |
| CXCR3 | Neutrophil_TIMER | **0.187** | **0.0005** | **0.002** |
| CXCL9 | Neutrophil_TIMER | **0.318** | **1.49E-09** | **1.43E-08** |
| CXCL10 | Neutrophil_TIMER | **0.213** | **6.94E-05** | **0.0004** |
| CXCL11 | Neutrophil_TIMER | **0.265** | **5.84E-07** | **3.87E-06** |
| CXCR4 | Neutrophil_TIMER | **0.400** | **1.08E-14** | **1.52E-13** |
| CXCR7 | Neutrophil_TIMER | **0.323** | **7.71E-10** | **2.01E-08** |
| CXCL12 | Neutrophil_TIMER | **0.182** | **0.001** | **0.004** |
| CXCR5 | Neutrophil_TIMER | **0.218** | **4.52E-05** | **0.0003** |
| CXCL13 | Neutrophil_TIMER | **0.179** | **0.001** | **0.004** |
| CXCR6 | Neutrophil_TIMER | **0.208** | **9.62E-05** | **0.001** |
| CXCL16 | Neutrophil_TIMER | 0.099 | 0.066 | 0.122 |
| XCR1 | Neutrophil_TIMER | **0.232** | **1.36E-05** | **7.58E-05** |
| XCL1 | Neutrophil_TIMER | **0.130** | **0.015** | **0.061** |
| XCL2 | Neutrophil_TIMER | 0.097 | 0.073 | 0.170 |
| CX3CR1 | Neutrophil_TIMER | **0.290** | **4.25E-08** | **4.07E-07** |
| CX3CL1 | Neutrophil_TIMER | **0.133** | **0.013** | **0.041** |

The bold text means positive correlation (Spearman’s *p* < 0.05, *p* > 0), the symbol # means negative correlation (Spearman’s *p* < 0.05, *p* < 0), the others mean not significant (Spearman’s *p* > 0.05).

Supplementary Table 6. The relationship between HCC-associated chemokine expression and infiltrate estimation value for macrophages in the TCGA LIHC dataset

| HCC‑associated chemokines | infiltrates estimation  value | rho | *p* | adjustment *p* |
| --- | --- | --- | --- | --- |
| CCR1 | Macrophage_TIMER | **0.529** | **2.81E-26** | **1.03E-25** |
| CCL3 | Macrophage_TIMER | **0.227** | **2.09E-05** | **3.94E-05** |
| CCL5 | Macrophage_TIMER | **0.299** | **1.56E-08** | **3.25E-08** |
| CCL14 | Macrophage_TIMER | -0.133# | 0.013# | 0.033# |
| CCL23 | Macrophage_TIMER | **0.176** | **0.001** | **0.002** |
| CCR2 | Macrophage_TIMER | **0.364** | **2.97E-12** | **8.83E-12** |
| CCL2 | Macrophage_TIMER | **0.362** | **4.27E-12** | **1.76E-11** |
| CCR3 | Macrophage_TIMER | **0.276** | **1.80E-07** | **9.83E-07** |
| CCL24 | Macrophage_TIMER | 0.072 | 0.181 | 0.275 |
| CCR4 | Macrophage_TIMER | **0.375** | **6.18E-13** | **2.69E-12** |
| CCL17 | Macrophage_TIMER | **0.239** | **7.15E-06** | **3.73E-05** |
| CCL22 | Macrophage_TIMER | **0.325** | **6.27E-10** | **2.58E-09** |
| CCR5 | Macrophage_TIMER | **0.450** | **1.29E-18** | **3.57E-18** |
| CCL4 | Macrophage_TIMER | **0.346** | **3.85E-11** | **9.48E-11** |
| CCR6 | Macrophage_TIMER | **0.294** | **2.67E-08** | **1.36E-07** |
| CCL20 | Macrophage_TIMER | **0.221** | **3.38E-05** | **0.0001** |
| CCR7 | Macrophage_TIMER | **0.222** | **3.14E-05** | **6.56E-05** |
| CCL19 | Macrophage_TIMER | **0.121** | **0.024** | **0.041** |
| CCR8 | Macrophage_TIMER | **0.370** | **1.19E-12** | **4.40E-12** |
| CCL1 | Macrophage_TIMER | -0.023 | 0.669 | 0.775 |
| CCR9 | Macrophage_TIMER | **0.132** | **0.014** | **0.039** |
| CCL25 | Macrophage_TIMER | -0.020 | 0.710 | 0.817 |
| CCR10 | Macrophage_TIMER | **0.299** | **1.56E-08** | **3.61E-07** |
| CCL27 | Macrophage_TIMER | 0.093 | 0.085 | 0.357 |
| CCL28 | Macrophage_TIMER | **0.411** | **1.78E-15** | **2.67E-13** |
| CXCR1 | Macrophage_TIMER | **0.166** | **0.002** | **0.009** |
| CXCL6 | Macrophage_TIMER | **0.342** | **7.12E-11** | **3.88E-09** |
| CXCR2 | Macrophage_TIMER | **0.270** | **3.64E-07** | **2.01E-06** |
| CXCL1 | Macrophage_TIMER | **0.325** | **6.16E-10** | **7.25E-09** |
| CXCL2 | Macrophage_TIMER | 0.026 | 0.636 | 0.735 |
| CXCL3 | Macrophage_TIMER | **0.345** | **4.47E-11** | **6.54E-10** |
| CXCL5 | Macrophage_TIMER | **0.289** | **4.88E-08** | **5.05E-07** |
| CXCL8 | Macrophage_TIMER | **0.343** | **5.53E-11** | **6.56E-10** |
| CXCR3 | Macrophage_TIMER | **0.316** | **1.99E-09** | **4.88E-09** |
| CXCL9 | Macrophage_TIMER | **0.209** | **9.02E-05** | **0.0002** |
| CXCL10 | Macrophage_TIMER | **0.143** | **0.008** | **0.011** |
| CXCL11 | Macrophage_TIMER | **0.190** | **0.000** | **0.001** |
| CXCR4 | Macrophage_TIMER | **0.544** | **6.25E-28** | **9.15E-27** |
| CXCR7 | Macrophage_TIMER | **0.208** | **0.0001** | **0.0006** |
| CXCL12 | Macrophage_TIMER | **0.256** | **1.39E-06** | **5.22E-06** |
| CXCR5 | Macrophage_TIMER | **0.252** | **2.13E-06** | **6.99E-06** |
| CXCL13 | Macrophage_TIMER | **0.115** | **0.032** | **0.048** |
| CXCR6 | Macrophage_TIMER | **0.332** | **2.65E-10** | **5.97E-10** |
| CXCL16 | Macrophage_TIMER | **0.274** | **2.32E-07** | **6.29E-07** |
| XCR1 | Macrophage_TIMER | **0.214** | **6.31E-05** | **0.0002** |
| XCL1 | Macrophage_TIMER | **0.255** | **1.57E-06** | **5.45E-06** |
| XCL2 | Macrophage_TIMER | **0.225** | **2.38E-05** | **4.83E-05** |
| CX3CR1 | Macrophage_TIMER | **0.336** | **1.58E-10** | **9.45E-10** |
| CX3CL1 | Macrophage_TIMER | **0.254** | **1.82E-06** | **1.27E-05** |

The bold text means positive correlation (Spearman’s *p* < 0.05, *p* > 0), the symbol # means negative correlation (Spearman’s *p* < 0.05, *p* < 0), the others mean not significant (Spearman’s *p* > 0.05).

Supplementary Table 7. The relationship between HCC-associated chemokine expression and infiltrate estimation value for dendritic cells in the TCGA LIHC dataset

| chemokines |  | | | |
| --- | --- | --- | --- | --- |
| CCR1 | Myeloid dendritic | **0.596** | **1.27E-34** | **1.36E-33** |
|  | cell_TIMER |  |  |  |
| CCL3 | Myeloid dendritic | **0.435** | **2.28E-17** | **1.75E-16** |
|  | cell_TIMER |  |  |  |
| CCL5 | Myeloid dendritic | **0.504** | **1.29E-23** | **6.75E-23** |
|  | cell_TIMER |  |  |  |
| CCL14 | Myeloid dendritic | -0.363# | 3.44E-12# | 5.70E-11# |
|  | cell_TIMER |  |  |  |
| CCL23 | Myeloid dendritic | **0.119** | **0.028** | **0.052** |
|  | cell_TIMER |  |  |  |
| CCR2 | Myeloid dendritic | **0.523** | **1.23E-25** | **7.44E-25** |
|  | cell_TIMER |  |  |  |
| CCL2 | Myeloid dendritic | **0.413** | **1.17E-15** | **8.61E-15** |
|  | cell_TIMER |  |  |  |
| CCR3 | Myeloid dendritic | **0.351** | **2.04E-11** | **2.08E-10** |
|  | cell_TIMER |  |  |  |
| CCL24 | Myeloid dendritic | **0.218** | **4.56E-05** | **0.0003** |
|  | cell_TIMER |  |  |  |
| CCR4 | Myeloid dendritic | **0.439** | **1.10E-17** | **5.82E-17** |
|  | cell_TIMER |  |  |  |
| CCL17 | Myeloid dendritic | **0.300** | **1.25E-08** | **5.24E-08** |
|  | cell_TIMER |  |  |  |
| CCL22 | Myeloid dendritic | **0.454** | **6.32E-19** | **3.35E-18** |
|  | cell_TIMER |  |  |  |
| CCR5 | Myeloid dendritic | **0.670** | **3.29E-46** | **2.67E-45** |
|  | cell_TIMER |  |  |  |
| CCL4 | Myeloid dendritic | **0.561** | **5.34E-30** | **4.34E-29** |
|  | cell_TIMER |  |  |  |
| CCR6 | Myeloid dendritic | **0.410** | **2.17E-15** | **1.29E-14** |
|  | cell_TIMER |  |  |  |
| CCL20 | Myeloid dendritic | **0.326** | **5.47E-10** | **7.51E-09** |
|  | cell_TIMER |  |  |  |
| CCR7 | Myeloid dendritic | **0.419** | **4.41E-16** | **2.06E-15** |
|  | cell_TIMER |  |  |  |
| CCL19 | Myeloid dendritic | **0.306** | **6.16E-09** | **2.08E-08** |
|  | cell_TIMER |  |  |  |
| CCR8 | Myeloid dendritic | **0.550** | **1.11E-28** | **9.85E-28** |
|  | cell_TIMER |  |  |  |
| CCL1 | Myeloid dendritic | **0.181** | **0.001** | **0.003** |
|  | cell_TIMER |  |  |  |
| CCR9 | Myeloid dendritic | **0.141** | **0.009** | **0.028** |
|  | cell_TIMER |  |  |  |
| CCL25 | Myeloid dendritic | -0.019 | 0.727 | 0.843 |
|  | cell_TIMER |  |  |  |
| CCR10 | Myeloid dendritic | **0.407** | **3.57E-15** | **2.03E-13** |
|  | cell_TIMER |  |  |  |
| CCL27 | Myeloid dendritic | **0.130** | **0.016** | **0.167** |
|  | cell_TIMER |  |  |  |
| CCL28 | Myeloid dendritic | **0.281** | **1.06E-07** | **4.22E-06** |
|  | cell_TIMER |  |  |  |
| CXCR1 | Myeloid dendritic | **0.114** | **0.034** | **0.110** |
|  | cell_TIMER |  |  |  |
| CXCL6 | Myeloid dendritic | **0.309** | **4.32E-09** | **1.07E-07** |
|  | cell_TIMER |  |  |  |
| CXCR2 | Myeloid dendritic | **0.239** | **7.27E-06** | **5.08E-05** |
|  | cell_TIMER |  |  |  |
| CXCL1 | Myeloid dendritic | **0.346** | **3.88E-11** | **7.01E-10** |
|  | cell_TIMER |  |  |  |
| CXCL2 | Myeloid dendritic | -0.001 | 0.980 | 0.997 |
|  | cell_TIMER |  |  |  |

HCC‑associated infiltrates estimation value rho *p* adjustment *p*

| CXCL3 | Myeloid dendritic | **0.440** | **9.13E-18** | **6.06E-16** |
| --- | --- | --- | --- | --- |
|  | cell_TIMER |  |  |  |
| CXCL5 | Myeloid dendritic | **0.345** | **4.38E-11** | **9.38E-10** |
|  | cell_TIMER |  |  |  |
| CXCL8 | Myeloid dendritic | **0.382** | **1.92E-13** | **5.10E-12** |
|  | cell_TIMER |  |  |  |
| CXCR3 | Myeloid dendritic | **0.537** | **4.11E-27** | **2.55E-26** |
|  | cell_TIMER |  |  |  |
| CXCL9 | Myeloid dendritic | **0.394** | **3.10E-14** | **1.18E-13** |
|  | cell_TIMER |  |  |  |
| CXCL10 | Myeloid dendritic | **0.247** | **3.36E-06** | **8.35E-06** |
|  | cell_TIMER |  |  |  |
| CXCL11 | Myeloid dendritic | **0.315** | **2.24E-09** | **9.02E-09** |
|  | cell_TIMER |  |  |  |
| CXCR4 | Myeloid dendritic | **0.649** | **1.06E-42** | **4.67E-41** |
|  | cell_TIMER |  |  |  |
| CXCR7 | Myeloid dendritic | **0.211** | **7.66E-05** | **0.001** |
|  | cell_TIMER |  |  |  |
| CXCL12 | Myeloid dendritic | **0.315** | **2.28E-09** | **1.57E-08** |
|  | cell_TIMER |  |  |  |
| CXCR5 | Myeloid dendritic | **0.469** | **2.78E-20** | **1.94E-19** |
|  | cell_TIMER |  |  |  |
| CXCL13 | Myeloid dendritic | **0.287** | **5.89E-08** | **2.07E-07** |
|  | cell_TIMER |  |  |  |
| CXCR6 | Myeloid dendritic | **0.530** | **2.38E-26** | **1.24E-25** |
|  | cell_TIMER |  |  |  |
| CXCL16 | Myeloid dendritic | **0.306** | **6.74E-09** | **3.89E-08** |
|  | cell_TIMER |  |  |  |
| XCR1 | Myeloid dendritic | **0.276** | **1.80E-07** | **8.86E-07** |
|  | cell_TIMER |  |  |  |
| XCL1 | Myeloid dendritic | **0.429** | **7.48E-17** | **8.76E-16** |
|  | cell_TIMER |  |  |  |
| XCL2 | Myeloid dendritic | **0.427** | **9.57E-17** | **5.60E-16** |
|  | cell_TIMER |  |  |  |
| CX3CR1 | Myeloid dendritic | **0.377** | **4.17E-13** | **3.60E-12** |
|  | cell_TIMER |  |  |  |
| CX3CL1 | Myeloid dendritic | **0.360** | **5.12E-12** | **8.31E-11** |
|  | cell_TIMER |  |  |  |

The bold text means positive correlation (Spearman’s *p* < 0.05, *p* > 0), the symbol # means negative correlation (Spearman’s *p* < 0.05, *p* < 0), the others mean not significant (Spearman’s *p* > 0.05).

Supplementary Table 8. The relationship between HCC-associated chemokine expression and immune infiltrate estimation values in the TCGA LIHC dataset

HCC‑associated

chemokines

T cell CD8+

T cell CD4+ B cell Neutrophi

l

Macrophag e

Myeloid dendritic cell

| CCR1 | **0.494** | 0.086 | **0.241** | **0.376** | **0.529** | **0.596** |
| --- | --- | --- | --- | --- | --- | --- |
| CCL3 | **0.419** | -0.105 | **0.169** | **0.233** | **0.227** | **0.435** |
| CCL5 | **0.578** | **0.131** | **0.345** | **0.239** | **0.299** | **0.504** |
| CCL14 | 0.048 | -0.046 | -0.291# | -0.198# | -0.133# | -0.363# |
| CCL23 | **0.250** | 0.065 | 0.002 | 0.069 | **0.176** | **0.119** |
| CCR2 | **0.449** | **0.270** | **0.366** | **0.261** | **0.364** | **0.523** |
| CCL2 | **0.292** | **0.172** | **0.158** | **0.288** | **0.362** | **0.413** |
| CCR3 | **0.152** | 0.082 | **0.171** | **0.338** | **0.276** | **0.351** |
| CCL24 | **0.217** | 0.048 | **0.127** | 0.088 | 0.072 | **0.218** |
| CCR4 | **0.400** | **0.234** | **0.305** | **0.328** | **0.375** | **0.439** |
| CCL17 | **0.212** | **0.270** | **0.192** | **0.118** | **0.239** | **0.300** |
| CCL22 | **0.268** | **0.371** | **0.294** | **0.237** | **0.325** | **0.454** |
| CCR5 | **0.542** | **0.295** | **0.462** | **0.299** | **0.450** | **0.670** |
| CCL4 | **0.589** | -0.044 | **0.281** | **0.308** | **0.346** | **0.561** |
| CCR6 | 0.026 | **0.479** | **0.378** | 0.087 | **0.294** | **0.410** |
| CCL20 | 0.001 | **0.284** | **0.229** | **0.118** | **0.221** | **0.326** |
| CCR7 | **0.403** | **0.256** | **0.309** | **0.266** | **0.222** | **0.419** |
| CCL19 | **0.255** | **0.256** | **0.250** | **0.116** | **0.121** | **0.306** |
| CCR8 | **0.388** | **0.234** | **0.337** | **0.312** | **0.370** | **0.550** |
| CCL1 | 0.034 | **0.234** | **0.161** | **0.108** | -0.023 | **0.181** |
| CCR9 | **0.196** | **0.185** | 0.080 | **0.181** | **0.132** | **0.141** |
| CCL25 | 0.065 | 0.079 | 0.097 | -0.178# | -0.020 | -0.019 |
| CCR10 | 0.091 | **0.287** | **0.243** | **0.261** | **0.299** | **0.407** |
| CCL27 | 0.038 | 0.044 | 0.064 | 0.017 | 0.093 | **0.130** |
| CCL28 | **0.168** | **0.227** | 0.055 | **0.178** | **0.411** | **0.281** |
| CXCR1 | **0.145** | 0.042 | -0.043 | **0.249** | **0.166** | **0.114** |
| CXCL6 | 0.091 | **0.165** | **0.128** | **0.203** | **0.342** | **0.309** |
| CXCR2 | **0.258** | 0.059 | 0.046 | **0.364** | **0.270** | **0.239** |
| CXCL1 | 0.087 | **0.248** | **0.170** | **0.251** | **0.325** | **0.346** |
| CXCL2 | 0.083 | -0.028 | -0.009 | -0.018 | 0.026 | -0.001 |
| CXCL3 | **0.162** | **0.246** | **0.249** | **0.293** | **0.345** | **0.440** |
| CXCL5 | 0.038 | **0.206** | **0.161** | **0.216** | **0.289** | **0.345** |
| CXCL8 | **0.141** | **0.156** | **0.145** | **0.319** | **0.343** | **0.382** |
| CXCR3 | **0.446** | **0.279** | **0.423** | **0.187** | **0.316** | **0.537** |
| CXCL9 | **0.505** | 0.024 | **0.343** | **0.318** | **0.209** | **0.394** |
| CXCL10 | **0.466** | -0.044 | **0.250** | **0.213** | **0.143** | **0.247** |
| CXCL11 | **0.496** | -0.057 | **0.242** | **0.265** | **0.190** | **0.315** |
| CXCR4 | **0.416** | **0.328** | **0.478** | **0.400** | **0.544** | **0.649** |
| CXCR7 | **0.113** | 0.062 | -0.015 | **0.323** | **0.208** | **0.211** |
| CXCL12 | **0.195** | **0.282** | **0.135** | **0.182** | **0.256** | **0.315** |
| CXCR5 | **0.286** | **0.347** | **0.435** | **0.218** | **0.252** | **0.469** |
| CXCL13 | **0.258** | 0.072 | **0.252** | **0.179** | **0.115** | **0.287** |
| CXCR6 | **0.572** | **0.190** | **0.428** | **0.208** | **0.332** | **0.530** |
| CXCL16 | **0.107** | **0.282** | **0.348** | 0.099 | **0.274** | **0.306** |
| XCR1 | **0.314** | **0.156** | **0.186** | **0.232** | **0.214** | **0.276** |
| XCL1 | **0.294** | **0.157** | **0.260** | **0.130** | **0.255** | **0.429** |
| XCL2 | **0.395** | 0.091 | **0.310** | 0.097 | **0.225** | **0.427** |
| CX3CR1 | **0.282** | **0.220** | **0.190** | **0.290** | **0.336** | **0.377** |
| CX3CL1 | **0.215** | **0.223** | **0.244** | **0.133** | **0.254** | **0.360** |

The bold text means positive correlation (Spearman’s *p* < 0.05, *p* > 0), the symbol # means negative correlation (Spearman’s *p* < 0.05, *p* < 0), the others mean not significant (Spearman’s *p* > 0.05).
